# The SARS-CoV-2 and other human coronavirus spike proteins are fine-tuned towards temperature and proteases of the human airways

**DOI:** 10.1371/journal.ppat.1009500

**Published:** 2021-04-22

**Authors:** Manon Laporte, Valerie Raeymaekers, Ria Van Berwaer, Julie Vandeput, Isabel Marchand-Casas, Hendrik-Jan Thibaut, Dominique Van Looveren, Katleen Martens, Markus Hoffmann, Piet Maes, Stefan Pöhlmann, Lieve Naesens, Annelies Stevaert

**Affiliations:** 1 KU Leuven, Department of Microbiology, Immunology and Transplantation, Laboratory of Virology and Chemotherapy, Rega Institute, Leuven, Belgium; 2 KU Leuven, Department of Microbiology, Immunology and Transplantation, Laboratory of Virology and Chemotherapy, Translational Platform Virology and Chemotherapy, Rega Institute, Leuven, Belgium; 3 KU Leuven, Department of Microbiology, Immunology and Transplantation, Allergy and Clinical Immunology Research Unit, Leuven, Belgium; 4 German Primate Center–Leibniz Institute for Primate Research, Infection Biology Unit, Göttingen, Germany; 5 University Göttingen, Faculty of Biology and Psychology, Göttingen, Germany; 6 KU Leuven, Department of Microbiology, Immunology and Transplantation, Laboratory of Clinical and Epidemiological Virology, Rega Institute, Leuven, Belgium; Erasmus Medical Center, NETHERLANDS

## Abstract

The high transmissibility of SARS-CoV-2 is related to abundant replication in the upper airways, which is not observed for the other highly pathogenic coronaviruses SARS-CoV and MERS-CoV. We here reveal features of the coronavirus spike (S) protein, which optimize the virus towards the human respiratory tract. First, the S proteins exhibit an intrinsic temperature preference, corresponding with the temperature of the upper or lower airways. Pseudoviruses bearing the SARS-CoV-2 spike (SARS-2-S) were more infectious when produced at 33°C instead of 37°C, a property shared with the S protein of HCoV-229E, a common cold coronavirus. In contrast, the S proteins of SARS-CoV and MERS-CoV favored 37°C, in accordance with virus preference for the lower airways. Next, SARS-2-S-driven entry was efficiently activated by not only TMPRSS2, but also the TMPRSS13 protease, thus broadening the cell tropism of SARS-CoV-2. Both proteases proved relevant in the context of authentic virus replication. TMPRSS13 appeared an effective spike activator for the virulent coronaviruses but not the low pathogenic HCoV-229E virus. Activation of SARS-2-S by these surface proteases requires processing of the S1/S2 cleavage loop, in which both the furin recognition motif and extended loop length proved critical. Conversely, entry of loop deletion mutants is significantly increased in cathepsin-rich cells. Finally, we demonstrate that the D614G mutation increases SARS-CoV-2 stability, particularly at 37°C, and, enhances its use of the cathepsin L pathway. This indicates a link between S protein stability and usage of this alternative route for virus entry. Since these spike properties may promote virus spread, they potentially explain why the spike-G614 variant has replaced the early D614 variant to become globally predominant. Collectively, our findings reveal adaptive mechanisms whereby the coronavirus spike protein is adjusted to match the temperature and protease conditions of the airways, to enhance virus transmission and pathology.

## Author summary

The devastating COVID-19 pandemic is caused by SARS-CoV-2, a novel virus that despite recent zoonotic introduction is already very well adapted to its human host. Its rapid spread is related to abundant replication in the upper airways, which is not observed for other highly pathogenic human coronaviruses. To understand the role of the viral spike protein in this airway adaptation, we constructed pseudoviruses of SARS-CoV-2 and other coronaviruses that cause severe pneumonia or, on the contrary, a mild common cold. The key findings were verified with authentic virus. We reveal features of the spike proteins, which optimize the coronavirus towards specific parts of the respiratory tract. Namely, we show that the spike proteins exhibit intrinsic temperature preference to precisely match the upper (~33°C) or lower (37°C) airways. We recognized which proteases of human airways activate the spike for virus entry, in particular one protease that may mediate coronavirus virulence. Finally, a link was perceived between spike stability and entry via endosomal proteases. We propose that these mechanisms of spike fine-tuning may have contributed to a global shift in SARS-CoV-2 epidemiology, from the early spike-D614 to the currently predominating G614 variant.

## Introduction

The devastating COVID-19 pandemic is caused by the novel SARS-CoV-2 virus. Despite its recent zoonotic introduction in humans, this coronavirus (CoV) is already very well adapted for efficient respiratory droplet transmission and high-titer replication in human airways [1,2]. Its disease spectrum varies from mild respiratory symptoms to severe pneumonia [3], depending mostly on the patient’s age and comorbidities. The SARS-CoV-2 pandemic was preceded by local outbreaks of SARS-CoV and MERS-CoV, two other highly virulent viruses with a zoonotic origin [4,5]. Compared to SARS-CoV-2, SARS-CoV and MERS-CoV have far lower tropism for the upper respiratory tract [1,6]. In contrast, a mild common cold-like disease is typical for the endemic human CoVs 229E, NL63, OC43 and HKU1 [7]. Their zoonotic spillover probably occurred long time ago [8–10], implying extensive adaptation to the upper respiratory tract in which these common cold viruses are flourishing.

The efficient replication of SARS-CoV-2 all through the airway tract implicates that the virus is compatible with the temperature in its compartments, which evolves from ~30–32°C in the nose to 37°C in the deeper airways [11,12]. We recently showed that the hemagglutinin of influenza B virus has an intrinsic preference for 33°C to be robustly expressed, consistent with 33°C being the best temperature to propagate this virus. Other temperature profiles were recognized for the hemagglutinin proteins of human and avian influenza A viruses [13]. This subtle adaptation of viral glycoproteins to the temperature in the host organs might also apply to other respiratory viruses with a zoonotic origin, in particular CoVs. Since SARS-CoV-2 exhibits abundant replication in the nose [14], it is conceivable that its spike (S) protein is fine-tuned towards this compartment.

Within its first year of human circulation, SARS-CoV-2 has diverged into several variants, bearing mainly changes in the spike protein. Mutation D614G was already detected during the early phase of the pandemic and, after four months, the S^G614^ variant became globally predominant [15]. In humans [16] and in animal models [17,18], the S^G614^ variant generates slightly higher viral loads in the upper airways, which accords with higher transmissibility [17,19]. The D614G mutation was also shown to enhance virus replication in cultured airway epithelial cells [17–19] and increase infectivity of pseudovirus bearing SARS-CoV-2 S protein [19–23]. This has been attributed to higher protein stability; higher density in virus particles; increased adoption of the open spike conformation; or more efficient proteolytic activation of the S protein [16–20,22–26]. An explanation that reconciles these diverse observations is still lacking.

**Fig 1:**
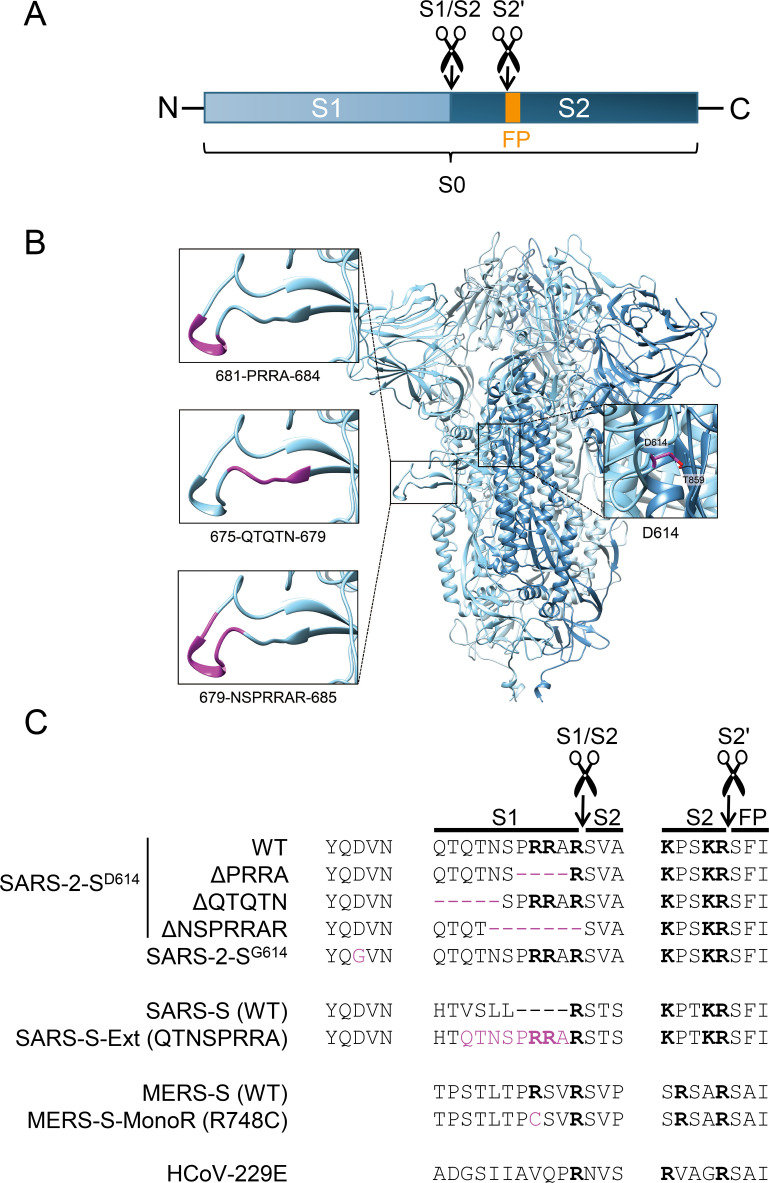
Study panel of wild-type and S1/S2 site mutant spikes, and the D614G mutant of SARS-2-S. (A) The CoV S protein contains two main cleavage sites: the S1/S2 site separates the S1 and S2 subunits, whereas S2′ cleavage liberates the fusion peptide (FP). (B) Structure of the SARS-CoV-2 spike trimer, based on PDB 6ZGE [90], in which we modelled the cleavage loop using SWISS-MODEL [91]. The amino acids shown in magenta were substituted or deleted, to create three S1/S2 loop mutants. The inset on the right shows residue D614, which forms a hydrogen bond with residue T859 in the S2 subunit of another protomer [22,23]. (C) Amino acid sequences around the S1/S2 and S2′ cleavage sites of the CoV spikes and mutant forms created in this study. Basic Arg (R) and Lys (K) residues are shown in bold.

The trimeric spike protein carries an S1 domain, responsible for receptor binding, and S2 domain, which mediates fusion between the viral envelope and a cellular membrane [27]. To become membrane fusion-competent, the full-length spike protein (S0) needs to be cleaved at its S1/S2 and S2′ sites (Fig 1A) [28,29]. Cleavage at the S2′ site might be sufficient to trigger membrane fusion and is referred to as activation, since it releases the internal fusion peptide [30]. The host protease TMPRSS2 is a prominent player in SARS-CoV-2 entry [31], however also other proteases may be involved, potentially broadening the cell or tissue tropism of this virus. In mice, TMPRSS2 knockout reduced lung pathology from SARS-CoV and MERS-CoV, but since virus replication was not abolished, other proteases appeared to take over [32]. The Type II Transmembrane Serine Protease (TTSP) family, to which TMPRSS2 belongs, contains in total 18 proteases, many of which are expressed in human airways [13]. Two recent analyses with a subset of TTSPs identified TMPRSS13 as a second prominent activator of the SARS-CoV-2 S protein (subsequently referred to as SARS-2-S) [33,34].

Regarding cleavage at the S1/S2 site, SARS-2-S is so far unique in bearing an extended S1/S2 cleavage loop with a multibasic furin recognition motif (RRAR) (Fig 1B and 1C) [35]. This loop extension is not present in the S protein of other lineage B betacoronaviruses [36]. The multibasic motif is assumedly processed by furin-like proteases [37–40], but also other proteases have been proposed [41]. S1/S2 priming proved crucial for efficient SARS-2-S activation by TMPRSS2 and for viral entry in the airway epithelium Calu-3 cell model [37,38,42]. A minimal furin recognition motif is also present in the S protein of MERS-CoV (MERS-S) [43,44], but not the spike protein of SARS-CoV (SARS-S) [38]. On the other hand, neither of these three CoV S proteins needs S1/S2 priming to mediate entry into cells with high levels of the endo/lysosomal cathepsin B/L proteases, which activate the S protein after virus uptake by endocytosis [30,39]. For SARS-2-S, the determinants governing S1/S2 processing are still far from clear. Virus passaging in Vero cells commonly leads to substitutions or deletions in the S1/S2 cleavage loop [45–52]. In animal models, these viruses exhibit reduced transmission [53] or virulence [54], providing an explanation why severe mutations in the S1/S2 loop are only rarely detected in humans [48,53,55] (S1 and S2 Tables).

The aim of this study was to assess how SARS-2-S (variant S^D614^ or S^G614^) is fine-tuned towards the temperature and proteases of the airways; and how these properties compare to those of SARS-S, MERS-S and the S protein of the common cold virus HCoV-229E. By performing pseudovirus production at 33°C and 37°C, we revealed that each spike protein exhibits an intrinsic and distinct temperature preference, correlating with compatibility with the upper or lower airways. We next addressed how SARS-2-S driven entry is controlled by host proteases that cleave its extended S1/S2 loop or S2′ site. Hence, we studied the entry behavior of different SARS-2-S loop deletion mutants, and we assessed which of the 18 human TTSPs act as CoV spike activators. Finally, we compared the S^D614^ and S^G614^ SARS-CoV-2 variants in terms of temperature and protease dependency, to appreciate how these spike features might be linked to virus transmissibility.

## Results

### Wild-type and mutant S proteins in the study panel

Our panel of different S proteins included SARS-2-S, SARS-S and MERS-S, plus the S protein of HCoV-229E (229E-S). For SARS-2-S, we included the two variants bearing Asp (D) or Gly (G) at residue 614, which is located at an inter-protomer interface in the spike trimer (Fig 1B) [22,23]. Besides, to investigate the determinants and impact of S1/S2 priming in SARS-2-S, we generated three deletion mutants missing parts of the extended S1/S2 cleavage loop (Fig 1B and 1C). The ΔPRRA mutant lacks the furin cleavage motif (RRAR) and its cleavage site is identical to that of the SARS-CoV-2-related bat CoV RaTG13 [36]. The ΔQTQTN mutant lacks a sequence preceding the RRAR motif, while mutant ΔNSPRRAR lacks the RRAR motif plus three flanking amino acid residues. These or very similar deletions are commonly detected during passaging of SARS-CoV-2 in Vero cells (see S3 Table and references therein), suggesting that they might confer a growth advantage in this cell line. For comparison, we created a mutant form of SARS-S, bearing the SARS-2-S multibasic cleavage site and preceding residues, and we generated a mutant of MERS-S, in which the furin motif was destroyed (R748C) (Fig 1C).

### The spikes of SARS-CoV-2 and HCoV-229E prefer 33°C for pseudovirus production, while the SARS-CoV and MERS-CoV spikes prefer 37°C

**Fig 2:**
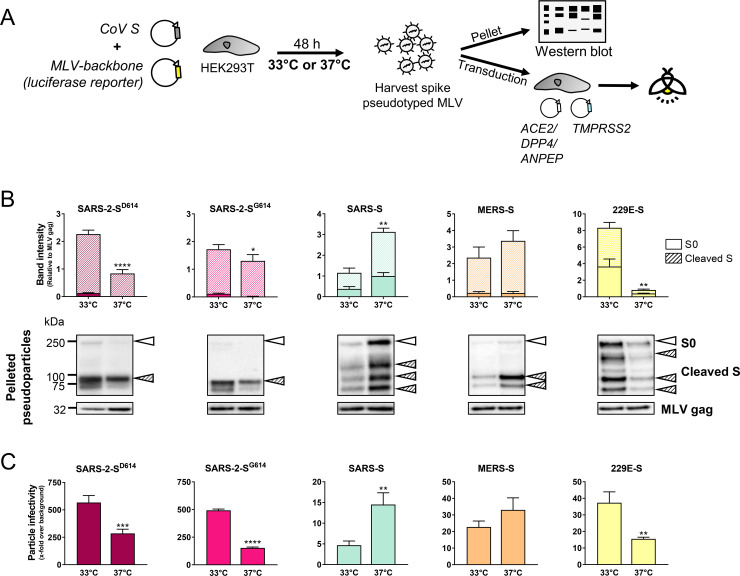
S protein level and infectivity of pseudovirions are dependent on the production temperature. (A) Experiment set-up. S-bearing pseudoviruses were produced in HEK293T cells at either 33°C or 37°C, and the released particles were pelleted to determine S content by western blot. In parallel, they were used to transduce HEK293T target cells expressing the appropriate receptor and TMPRSS2. (B) The graphs show S content relative to that of MLV-gag (mean ± SEM of four independently produced stocks). Representative blots show bands of uncleaved and cleaved S protein. (C) Particle infectivity was measured by luminescence read-out at day 3 post transduction (mean ± SEM of three independently produced stocks). *, P ≤ 0.05; **, P ≤ 0.01; ***, P ≤ 0.001; ****, P ≤ 0.0001 (two-tailed unpaired t-test; 37°C versus 33°C).

Considering that a temperature gradient exists in the human respiratory tract, we first investigated the influence of temperature on spike functioning. We hypothesized that such an effect might help to explain why the common cold virus HCoV-229E replicates more efficiently at 33°C and 35°C, when compared to 37°C and, particularly, 39°C (S1 Fig). To investigate the spike’s temperature dependency, we produced murine leukemia virus (MLV) particles bearing the S proteins of the highly pathogenic species SARS-CoV-2, SARS-CoV and MERS-CoV, or the common cold virus HCoV-229E, and production was performed in HEK293T cells at upper respiratory tract (URT) (33°C) or lower respiratory tract (LRT) (37°C) temperature (Fig 2A).

Western blot analysis revealed that the production temperature impacted S proteins levels in pelleted pseudoparticles, while MLV-gag levels were the same at 33°C and 37°C (Fig 2B). Also S protein cleavage by host cell proteases showed no difference (Fig 2B). Specifically, at 37°C, SARS-2-S levels were lower than at 33°C, and this temperature effect appeared slightly more pronounced for the S^D614^ (P<0.0001) than S^G614^ variant (P = 0.012). At each temperature, S protein levels were not significantly different when the two variants were compared. They also showed comparable S1/S2 cleavage efficiency, and both generated pseudovirions with S1/S2 pre-cleaved spikes. This contradicts another study [20] but agrees with two other reports [19,22]. The 33°C preference was even more apparent for 229E-S (= 10-fold higher level at 33°C than at 37°C; P = 0.0027). The picture was entirely opposite for SARS-S, where S-levels were 2.7-fold higher at 37°C than at 33°C (P = 0.0053), and a similar trend was observed for MERS-S.

Next, the particles were transduced into HEK293T cells transfected with TMPRSS2 and the appropriate virus receptor (Fig 2C). Whereas the signal was similar whether virus entry (i.e. target cell transduction) was performed at 33°C or 37°C (S2 Fig), the temperature used for pseudovirus production had a clear effect on particle infectivity (Fig 2C). In agreement with the western blot data, particles pseudotyped with SARS-2-S^D614^, SARS-2-S^G614^ and 229E-S showed significantly (P<0.01) higher infectivity when produced at 33°C instead of 37°C, but the reverse effect was observed for SARS-S and MERS-S. The influence of the production temperature was also seen with another pseudovirus system (S3 Fig). Using a vesicular stomatitis virus (VSV) backbone and BHK-21J producer cells, we confirmed that SARS-2-S- and 229E-S-bearing pseudoparticles showed higher infectivity when produced at 32°C, while SARS-S and MERS-S again favored 37°C.

Collectively, this indicates that S protein levels and infectivity of S-pseudotyped particles are dependent on the production temperature. The preference for 33°C proved strongest for 229E-S but was also significant for SARS-2-S. On the contrary, MERS-S and SARS-S prefer 37°C for pseudovirus production.

### Compared to SARS-CoV-2 virus bearing S^D614^, the S^G614^ variant appears slightly more stable at 37°C

**Fig 3:**
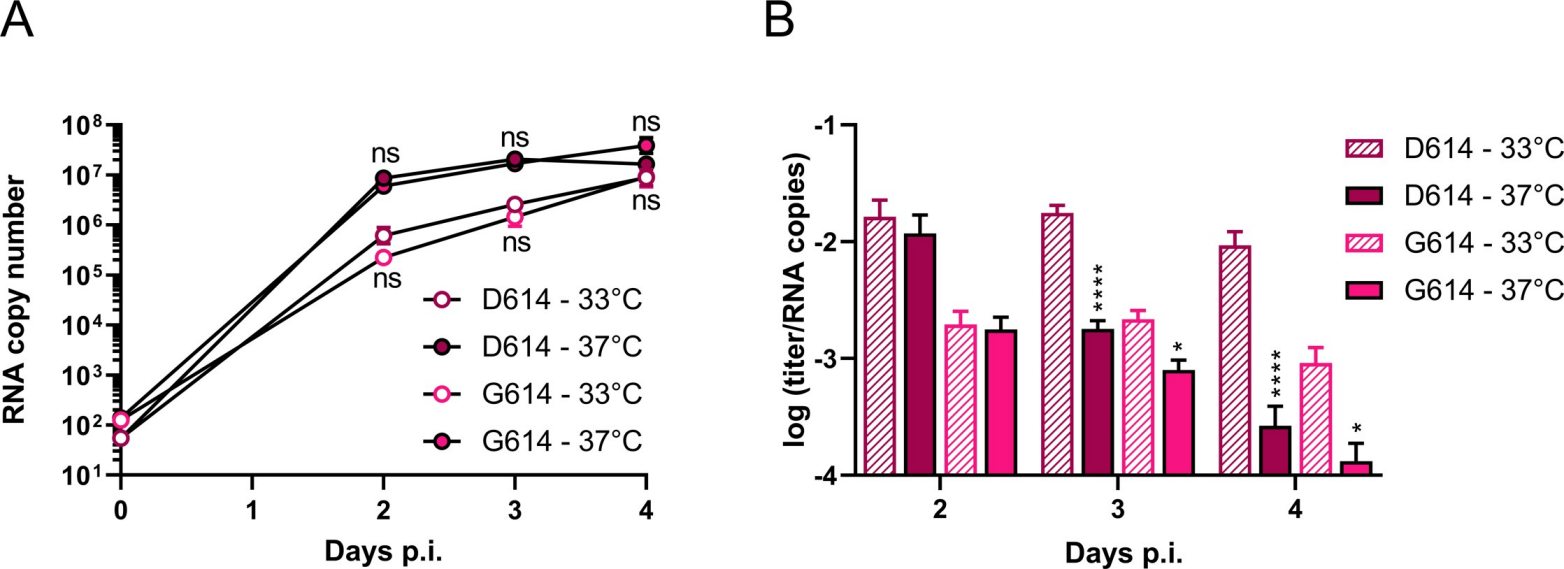
Temperature impacts particle infectivity of replicating SARS-CoV-2 virus. Calu-3 cells were infected with SARS-CoV-2 virus bearing variation S^D614^ or S^G614^, and incubated at 33°C or 37°C. (A) Number of viral genome copies in the supernatant, determined by RT-qPCR. (B) Infectivity of produced virus particles, expressed as the ratio of infectious titer (determined by virus titration in Vero E6 cells) over viral genome copy number. ns, P > 0.05; *, P ≤ 0.05; ****, P ≤ 0.0001 (two-tailed unpaired t-test; 37°C versus 33°C). Results are the mean ± SEM; N = 4, performed in duplicate.

To assess the effect of temperature on authentic SARS-CoV-2 virus replication and a possible effect of the D614G mutation, we used two SARS-CoV-2 virus strains (achieved as clinical isolates) bearing S^D614^ or S^G614^. At day 2, 3 and 4 post infection (p.i.) of Calu-3 cells, supernatants were harvested and analyzed for infectious virus titer and viral RNA copy number, the ratio of which represents the infectivity of the virions released in the culture medium (Fig 3). The two virus strains generated comparable RNA loads, and incubation at 37°C yielded higher RNA copy numbers compared to 33°C (Fig 3A). For both strains, virion infectivity remained largely stable at 33°C (Fig 3B). At 37°C, both viruses showed deterioration, but the decline was clearly faster for the S^D614^ strain. Specifically, when produced at 37°C, its infectivity was 10-fold (day 3 p.i.) to 35-fold (day 4 p.i.) lower than at 33°C (P < 0.0001), while the S^G614^ virus was only 2.7- to 7-fold less infectious at 37°C versus 33°C (P = 0.02). This provides indication that variation S^G614^ increases S protein stability at 37°C. Still, since our two virus strains contained also other differences than this one spike mutation, an influence of these other factors cannot be fully excluded.

In short, we observed a subtle yet significant effect of temperature on S-pseudotyped virus production and on stability of authentic SARS-CoV-2 virus.

### Cleavage of the SARS-2-S S1/S2 site is determined by the multibasic motif as well as the length of the cleavage loop

**Fig 4:**
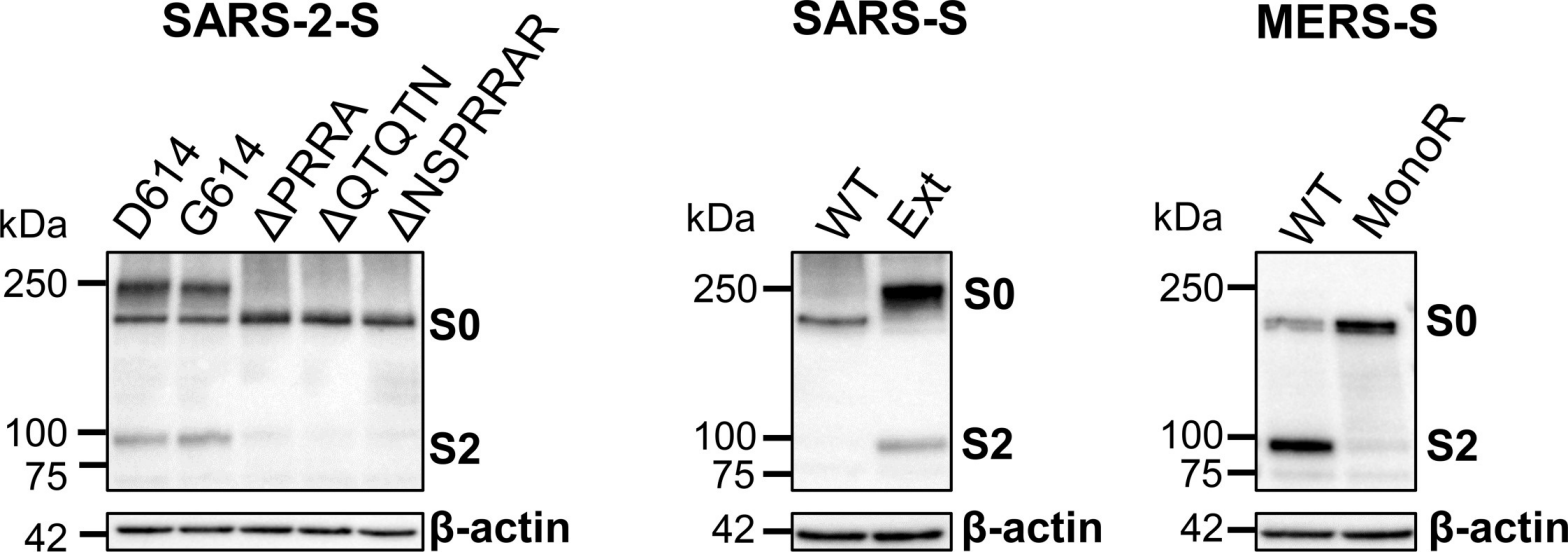
S1/S2 cleavage efficiency of WT and mutant spikes, expressed in HEK293T cells. The SARS-2-S, SARS-S and MERS-S proteins were expressed in HEK293T cells and at 48 h post transfection, extracts were made for western blot analysis of the V5-tagged proteins in uncleaved (S0) or S1/S2 cleaved form. β-Actin served as loading control.

Next, we investigated the impact of mutation D614G and of different alterations at the S1/S2 cleavage site (Fig 1C), on S priming and pseudovirus entry. First, we examined processing of S0 into S1/S2, in HEK293T cells transfected with the WT and mutant S protein forms (Fig 4). The S^D614^ and S^G614^ forms of SARS-2-S showed a strong S2 band, indicating equally efficient S1/S2 cleavage by one or more proteases expressed in these cells [37–40]. All three SARS-2-S^D614^ mutants bearing deletions in the S1/S2 loop showed virtually abrogated cleavage. The lack of cleavage for the ΔQTQTN mutant (which still possesses the multibasic furin motif but lacks preceding amino acids) indicates that not only the furin motif itself is critical for cleavage, but also the length of the loop presenting this motif. As expected [44], also WT MERS-S was efficiently cleaved, while its monobasic (monoR) cleavage site mutant was not processed. In contrast, WT SARS-S was barely cleaved, while proteolytic processing was efficient for the mutant containing the extended (Ext) S1/S2 cleavage loop of SARS-2-S, as anticipated [38].

### Loop deletion mutants of SARS-2-S show enhanced cathepsin-dependent entry, explaining their emergence in Vero cells

**Fig 5:**
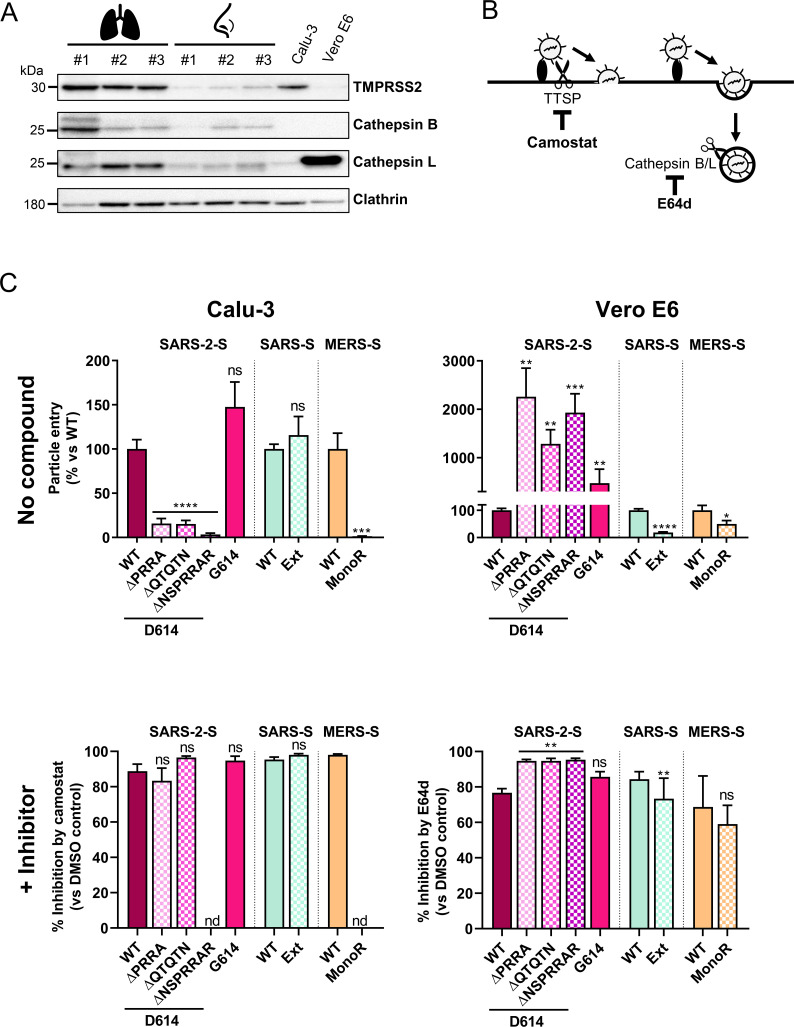
Entry of WT and S1/S2 mutant pseudoviruses in cells with different spike-activating proteases. (A) Western blot detection of TMPRSS2, cathepsin B and cathepsin L proteases in human lung tissue, human nasal tissue (each from three donors), Calu-3 cells and Vero E6 cells (loading control: clathrin). (B) The broad serine protease inhibitor camostat prevents fusion activation by TTSPs like TMPRSS2, whereas E64d inhibits cathepsin B/L-mediated fusion after virus uptake by endocytosis. (C) The graphs show entry efficiency (*top panels*) or % inhibition relative to the DMSO solvent control (*bottom panels*), by 50 μM camostat in Calu-3 cells (*left panel*) or 50 μM E64d in Vero E6 cells (*right panel*). ns, P > 0.05; *, P ≤ 0.05; **, P ≤ 0.01; ***, P ≤ 0.001, ****, P ≤ 0.0001 (unpaired two-tailed t-test; mutant forms versus WT). Results are the mean ± SEM from three experiments. nd, not determined.

Before conducting pseudovirus entry assays, we verified expression of the relevant proteases in the studied cell lines, i.e. Calu-3 cells, an epithelial cell line derived from a lung adenocarcinoma and Vero E6 cells, a cell line that is widely used for SARS-CoV-2 isolation and propagation. For comparison, we included samples of human nasal tissue and lung tissue, each from three different donors. As shown in Fig 5A, human nasal tissue and lung tissue were shown to contain TMPRSS2, cathepsin B and cathepsin L. Calu-3 cells express TMPRSS2 and a very low level of cathepsin L. The latter protease proved present at a very high level in Vero E6 cells. Besides, we verified expression of the viral receptors using RT-qPCR (S4 Fig). Human respiratory tissue contained the transcripts for angiotensin-converting enzyme 2 (ACE2; the entry receptor for SARS-CoV and SARS-CoV-2); dipeptidyl peptidase-4 (DPP4, the receptor for MERS-CoV); and aminopeptidase N (APN, the HCoV-229E receptor), and the mRNA levels were comparable for the two anatomic sites. *ACE2* and *DPP4*, but not *ANPEP/APN*, were expressed in Calu-3 and Vero E6 cells.

To evaluate how S1/S2 processing impacts virus entry into Calu-3 or Vero E6 cells, we used WT and mutant MLV pseudoviruses, produced at the optimal temperature established in the first part of this study. To discriminate the two S protein activation pathways, we included the protease inhibitors camostat and E64d (Fig 5B). All three SARS-2-S^D614^ mutants bearing deletions in the S1/S2 cleavage loop showed markedly reduced (6- to 30-fold; P < 0.0001 versus WT) entry into Calu-3 cells (Fig 5C, top left panel), in keeping with expectations [38,53]. Entry was fully rescued when exogenous trypsin was added during Calu-3 cell transduction (S5 Fig), indicating that the poor entry was due to a lack of S2′ cleavage by TTSPs, and not to inefficient receptor binding. Conversely, these three SARS-2-S loop deletion mutations resulted in 13- to 23-fold higher entry into Vero E6 cells, which depends on cathepsin L (Fig 5C, top right panel). This explains why SARS-CoV-2 passaging in Vero E6 cells regularly leads to emergence of viruses bearing substitutions or deletions in the S1/S2 loop [45–52].

Akin to SARS-2-S, mutant MERS-S pseudovirus bearing a monobasic (= non furin-cleavable) S1/S2 site showed dramatically reduced (71-fold) Calu-3 cell entry [43]. On the other hand, MERS-S driven entry into Vero E6 cells was not enhanced when its furin cleavability was abolished (i.e. 2-fold higher entry for WT than monobasic mutant; p = 0.04). For SARS-S pseudovirus, Calu-3 cell entry was unchanged when its S1/S2 sequence was exchanged for the extended loop of SARS-2-S, including the multibasic motif [38]. Entry into Vero E6 cells was more efficient (5-fold, P < 0.0001) for SARS-S-pseudovirus with WT protein (= short monobasic S1/S2 loop) than the mutant with extended multibasic loop.

Regarding mutation D614G in SARS-2-S, the S^G614^ form showed 4.7-fold higher entry into Vero E6 cells than the S^D614^ variant, while the difference in Calu-3 cells was only 1.5-fold and not significant (Fig 5C). This agrees with other reports showing 3- to 9-fold higher entry of the S^G614^ variant in pseudovirus assays [15,20,21,23,25].

Camostat produced >80% inhibition of pseudovirus entry into Calu-3 cells (Fig 5C, bottom left panel), corroborating that entry into these cells relies on serine proteases like TMPRSS2 [31]. In contrast, for all pseudoviruses, entry into Vero E6 cells was highly sensitive (59–95% inhibition) to E64d (Fig 5C, bottom right panel), confirming that S protein-driven entry into these cells is highly cathepsin L-dependent [28,31,56].

In summary, we demonstrate that, for SARS-2-S, not only the integrity of the furin motif at the S1/S2 site but also the length of the loop harboring this cleavage site, are required for S1/S2 priming. Mutants that cannot undergo this processing are boosted towards cathepsin-mediated entry, explaining why substitutions or deletions in the S1/S2 cleavage loop commonly emerge during SARS-CoV-2 propagation in Vero E6 cells that are rich in cathepsin L. Besides, these results underline the previously proposed concept [37–40,43] that SARS-2-S and MERS-S, but not SARS-S, require S1/S2 priming for TTSP-dependent entry into Calu-3 cells.

### SARS-CoV-2 bearing S^G614^ is more effective at using the cathepsin route

**Fig 6:**
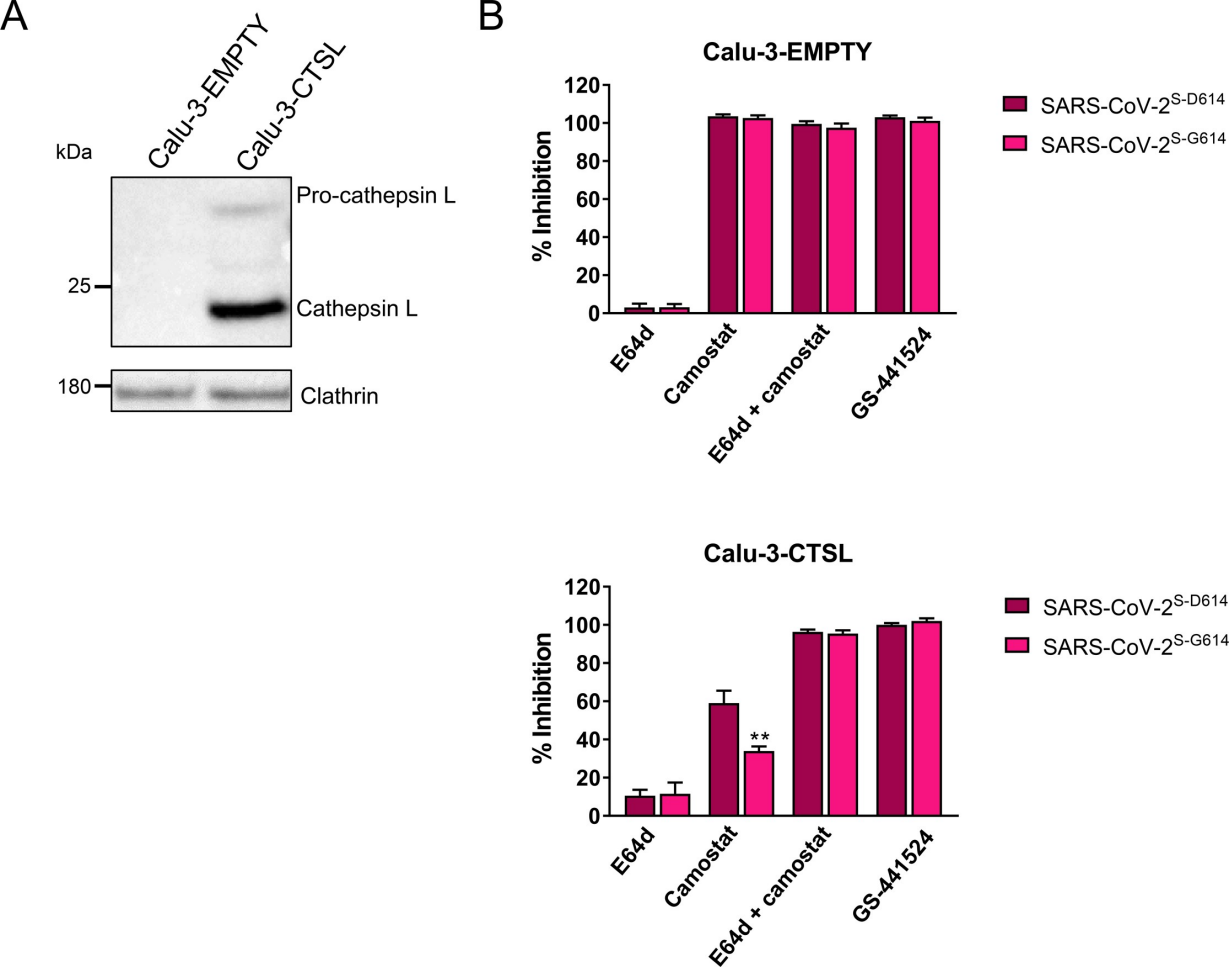
Virus bearing variation S^G614^ is more effective at using the cathepsin L route. Control (Calu-3-EMPTY) and cathepsin L-expressing (Calu-3-CTSL) Calu-3 cells were (A) checked for expression of cathepsin L and (B) infected with SARS-CoV-2 virus bearing spike variation D614 or G614, in the presence of 20 μM E64d; 100 μM camostat; 100 μM camostat plus 20 μM E64d; or 10 μM GS-441524. At 3 days p.i., the inhibitory effect of the compounds on virus replication was quantified by immunofluorescence for dsRNA. **, P ≤ 0.01 (unpaired two-tailed t-test; SARS-CoV-2^S-G614^ versus SARS-CoV-2^S-D614^). Results are the mean ± SEM from three experiments.

As mentioned above, the enhancing effect of SARS-2-S mutation D614G was higher in Vero E6 cells than in Calu-3 cells, suggesting that variation S^G614^ might boost in particular the cathepsin-dependent entry route. To investigate this further, we compared the two SARS-CoV-2 virus strains for their sensitivity to protease inhibitors in a control Calu-3 cell line (Calu-3-EMPTY) and in Calu-3 cells engineered to stably express cathepsin L (Calu-3-CTSL) (Fig 6A). The latter thus has the two entry routes–cathepsin-dependent and TTSP-dependent–available. As shown in Fig 6B, both strains were fully inhibited by camostat in Calu-3-EMPTY cells. In contrast, in Calu-3-CTSL cells, inhibition by camostat was clearly reduced, especially for the S^G614^ variant. Replication of the S^D614^ variant was significantly (P = 0.0016) more inhibited by camostat, i.e. by 59%, compared to 34% suppression for the S^G614^ variant. This indicates that the S^D614^ variant is more dependent on the TTSP route than the S^G614^ strain, suggesting that the S^G614^ variant is more efficient in using the redundant cathepsin-dependent pathway. For both viruses, adding E64d to camostat resulted in complete inhibition of virus replication. Both viruses were also fully suppressed, in both cell lines, by GS-441524, the nucleoside form of remdesivir. Combined with the above data from pseudovirus assays, this virus experiment provides evidence that mutation D614G increases entry of SARS-CoV-2 via the cathepsin route.

### Temperature and pH stability of the different spikes and mutants

**Fig 7:**
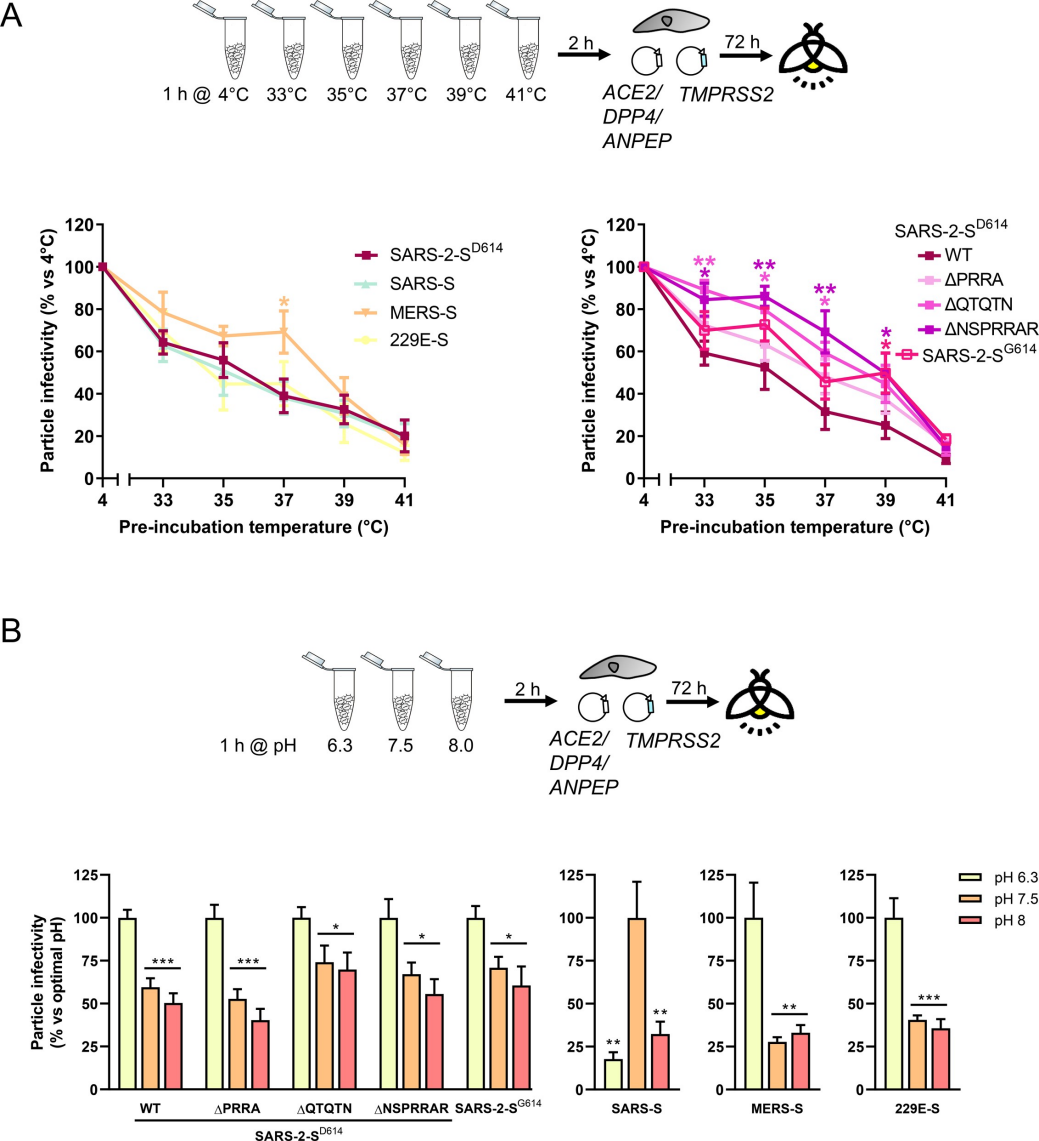
Temperature and pH stability of pseudovirions with WT or mutant S proteins. (A) The S-pseudotyped particles were incubated at the indicated temperatures for 1 h, followed by 2 h entry into HEK293T target cells and luminescence reading after 72 h. The Y-axis shows particle infectivity, relative to the condition incubated at 4°C (mean ± SEM, N = 3, performed in duplicate). *Left*: analysis of the four CoV pseudotypes; *right*: comparison of the S^D614^ and S^G614^ variants of SARS-2-S, and the three S1/S2 loop mutants.*, P ≤ 0.05; **, P ≤ 0.01 (Fisher’s LSD test; different strains or mutants versus SARS-2-S^D614^ at each temperature). (B) The pseudovirus stocks were adapted to pH 6.3, 7.5 or 8.0 and incubated for 1 h at 4°C. Next, their infectivity was determined in HEK293T target cells, as above. The Y-axis shows particle infectivity (mean ± SEM, N = 3, performed in triplicate), relative to the optimal pH condition (i.e. giving the highest luminescence signal). *, P ≤ 0.05; **, P ≤ 0.01; ***, P ≤ 0.001 (unpaired two-tailed t-test; versus optimal pH condition).

The above results showed that both temperature and different spike mutations impact pseudotype and virus infectivity. To examine whether this could be related to spike stability, we determined the thermostability of the various pseudoparticles. The pseudoviruses were incubated for 1 h at varying temperatures (range: 33 to 41°C, and 4°C for the control), then tested for infectivity in HEK293T cells expressing receptor and TMPRSS2 (Fig 7A). SARS-2-S^D614^ had comparable stability as SARS-S and 229E-S, while MERS-S appeared slightly more stable (Fig 7A, left panel). The thermostability of SARS-2-S^D614^ was increased when it was not cleaved at S1/S2; the stabilizing effect was particularly significant for the mutant in which most of the S1/S2 cleavage loop was deleted (ΔNSPRRAR; Fig 7A, right panel). Also substitution D614G generated a stabilizing effect, which was most pronounced at 39°C (P = 0.044). This concurs with a report that SARS-2-S^D614^ is less stable than SARS-2-S^G614^, possibly due to shedding of the S1 subunit [23].

Besides, we wondered whether the spike stability may be influenced by pH, considering that the human nasal cavity is slightly more acidic (pH ~6.3) than the lumen of human lungs (pH ~7.5). In addition, an increase in nasal pH (up to 8.3) is seen during respiratory infection [57]. Hence, we determined pseudovirus stability at pH 6.3, 7.5 and 8.0. Pseudovirus incubation (at 4°C) and infectivity testing were conducted similar as above (Fig 7B). For almost all pseudoviruses, pH 6.3 had a significant stabilizing effect compared to pH 7.5. No significant difference was seen between the pH 7.5 and 8.0 conditions. The one exception was SARS-S, which showed highest stability at pH 7.5, and significantly (P = 0.0014) lower infectivity after incubation at pH 6.3. The two variants of SARS-2-S had a similar pH profile.

### Among all 18 TTSPs, TMPRSS2 and TMPRSS13 are the best activators of SARS-2-S

**Fig 8:**
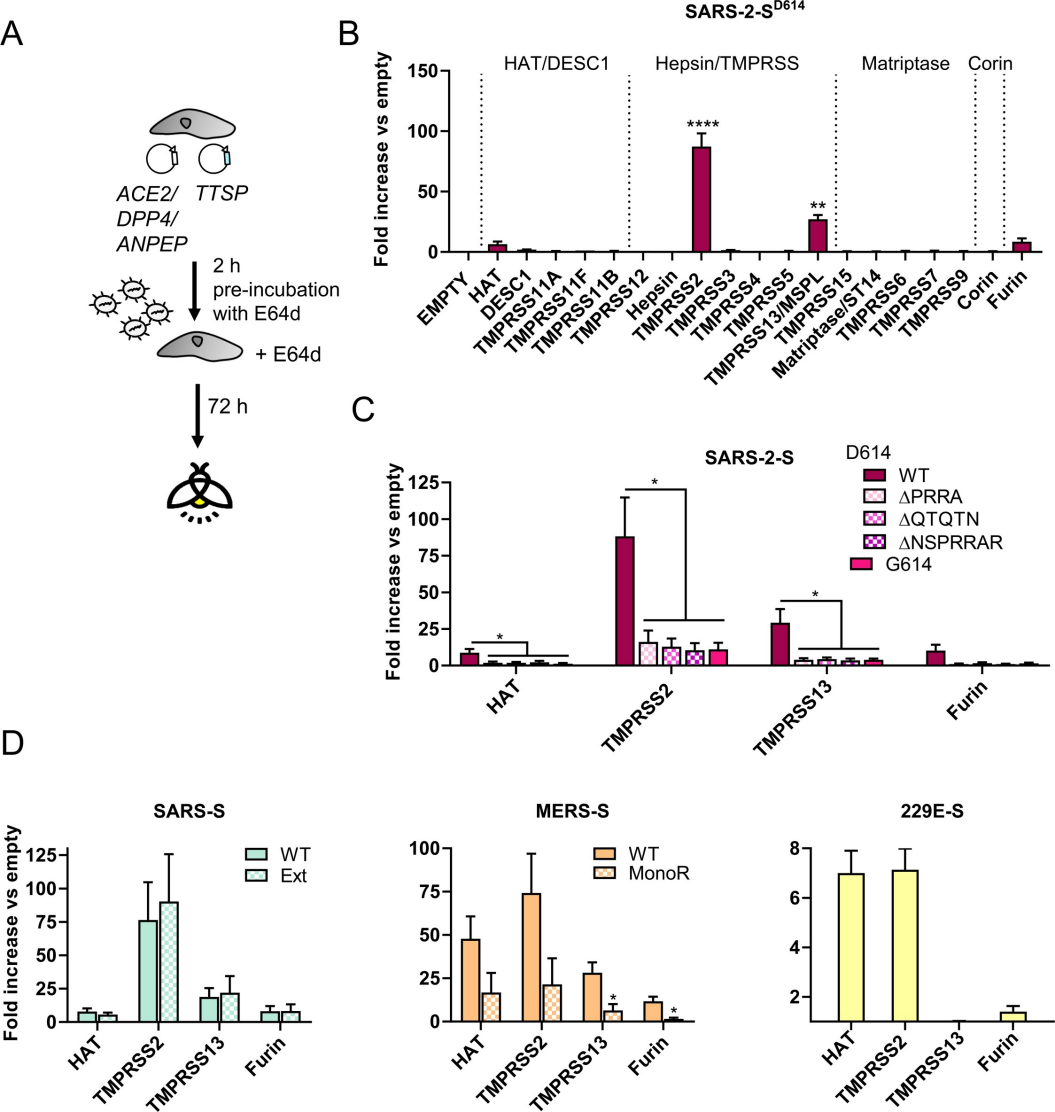
Activation of pseudovirus entry by different human TTSPs. (A) Experiment set-up. One day before transduction, HEK293T target cells were transfected with the appropriate receptor and one of the TTSPs. To block the cathepsin route, E64d was added at 2 h before and during transduction. (B) SARS-2-S activating capacity of the 18 human TTSPs. At the top of the graph, the four TTSP subfamilies are indicated. (C, D) The four TTSPs that proved active in panel B were evaluated for activation of wild-type and mutant forms of SARS-2-S (panel C), or SARS-S, MERS-S and 229E-S (panel D). An ordinary one-way ANOVA with Dunnett’s correction was used to analyze differences for empty versus TTSP plasmid conditions (panel B); and for SARS-2-S mutants versus D614 WT (panel C). Panel D: unpaired two-tailed t-test (WT versus mutant forms). *, P ≤ 0.05; **, P ≤ 0.01; ****, P ≤ 0.0001. Results are the mean ± SEM from three experiments.

Finally, we addressed whether, besides TMPRSS2, other TTSPs can activate SARS-2-S for virus entry. The pseudoviruses were applied to TTSP- plus receptor-transfected HEK293T cells in the presence of E64d, to shut off the parallel cathepsin route (Fig 8A). The expression plasmids that we used were previously shown to yield high protein levels for the various TTSPs [13,58]. We first investigated activation of SARS-2-S^D614^ by the 18 known human TTSPs or by furin (Fig 8B). The most efficient activator was TMPRSS2, followed by TMPRSS13 [also known as mosaic serine protease large-form (MSPL)], that was only 3-fold less effective. Human airway trypsin-like protease (HAT; also known as TMPRSS11D) and furin were, respectively, 13- and 10-fold less active than TMPRSS2. Mutating the S1/S2 cleavage loop abrogated activation by TMPRSS2, TMPRSS13, HAT and furin (Fig 8C), in accordance with the inability of these S proteins to mediate robust pseudovirus entry into Calu-3 cells. Also mutation D614G rendered SARS-2-S pseudovirus significantly less dependent on TTSP activation to enter HEK293T cells (Fig 8C). This nicely accords with the above finding that, compared to S^D614^ virus, the S^G614^ variant of SARS-CoV-2 is less sensitive to inhibition by camostat.

We next assessed whether these four proteases activate SARS-S, MERS-S and 229E-S (Fig 8D). TMPRSS2 activated the S proteins of all four CoVs, in keeping with published data [31,59–65]. Intriguingly, TMPRSS13 enhanced entry driven by the S proteins of the highly virulent SARS-CoV, SARS-CoV-2 and MERS-CoV (in line with other reports [33,34,58]), but not the common cold virus HCoV-229E. MERS-S and 229E-S were both activated by HAT, as reported earlier [63,66], with roughly the same efficiency as TMPRSS2. Finally, furin expression in the target cells gave weak activation of the four S proteins, which aligns with the report that extracellular furin can act at the stage of MERS-CoV entry [44]. The presence of an unaltered S1/S2 cleavage loop was required for efficient TTSP activation of SARS-2-S and MERS-S, as evident from the much lower activation of the S1/S2 mutants compared to the WT (Fig 8C and 8D). This effect may only apply to S proteins that naturally have a furin-cleavable S1/S2 site, since SARS-S showed equal activation by TTSPs whether the furin motif was present or not (compare WT and Ext mutant in Fig 8D).

**Fig 9:**
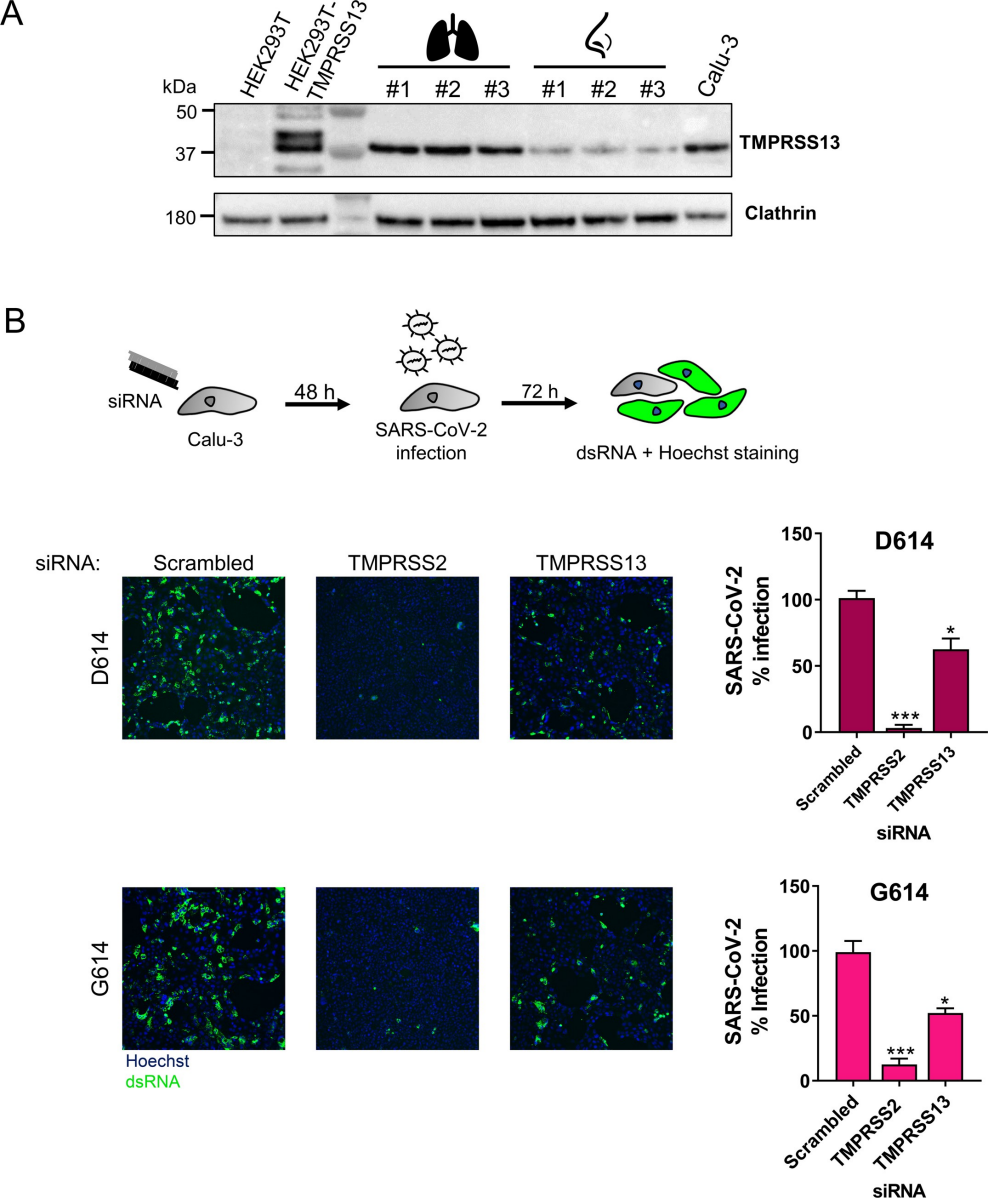
Knockdown of TMPRSS2 and TMPRSS13 reduces SARS-CoV-2 replication in Calu-3 cells. (A) Western blot analysis confirmed expression of TMPRSS13 in Calu-3 cells, and in human lung tissue and nasal tissue (each from three donors). HEK293T cells transfected with TMPRSS13 served as positive control. (B) Calu-3 cells were transfected with siRNA for TMPRSS2, TMPRSS13 or a scrambled control, and 48 h later infected with SARS-CoV-2 virus carrying S^D614^ or S^G614^. At 72 h p.i., virus replication was quantified by immunofluorescence for dsRNA; the pictures show representative images. The bar graphs show the number of infected cells, quantified by high-content imaging and expressed relative to the scrambled siRNA control. Results are the mean ± SEM (N = 3 with five or six replicates). *, P ≤ 0.05; ***, P ≤ 0.001 (one-way ANOVA, followed by Dunnett’s test; specific siRNA versus scrambled control).

Western blot analysis established that the active form of TMPRSS13 (having a MW of ~37 kDa [67]) is expressed in human lung [68] as well as nasal tissue (Fig 9A). Also Calu-3 cells were shown to express TMPRSS13 (Fig 9A). siRNA mediated-knockdown of TMPRSS13 in Calu-3 cells gave a significant reduction (about 40%, P<0.04; Fig 9B) in SARS-CoV-2 replication. Knockdown of TMPRSS2 served as positive control [37], and reduced virus levels by about 90% (P = 0.0007). Although less pronounced for TMPRSS13, this result provides definite evidence for the relevance of both proteases.

To summarize, these results corroborate TMPRSS2 as an efficient and broad S protein activator and TMPRSS13 as an activator of highly pathogenic CoVs.

## Discussion

In this study, we recognized two features of the SARS-CoV-2 spike protein, i.e. compatibility with the temperature gradient in the human respiratory tract and a well-tuned protease activation mechanism, which may well be two determinants for the high transmissibility and virulence of this virus. Our approach to include pseudoviruses with the spikes of SARS-CoV, MERS-CoV and HCoV-229E, offered the possibility to notice analogies and interpret our findings from a broader perspective.

First of all, we unveiled a distinct temperature preference for these different CoV spike proteins, that precisely matches the predilection of each virus for the upper or lower respiratory tract. Unlike SARS-CoV and MERS-CoV, but similar to common cold coronaviruses, SARS-CoV-2 replicates abundantly in the nose (~30–32°C) and upper airways [1,2], explaining its efficient transmission and mild URT disease in many infected persons. On the other hand, SARS-CoV-2 can also replicate in the lungs (37°C) to produce severe pathology, alike SARS-CoV and MERS-CoV. The parallel behavior of their spike proteins is striking: SARS-S and MERS-S were shown to favor 37°C for pseudovirus production, while SARS-2-S was found to prefer 33°C. This cooler temperature was also preferred by the spike protein of the common cold HCoV-229E virus. This subtle adaptation to the temperature of the upper or lower airways is also evident for influenza virus hemagglutinin [13], suggesting that it might be a commonality for human respiratory viruses. As to the biochemical basis, preference for 33°C for virus production indicates that the S protein is relatively unstable, since a cooler temperature should reduce conformational flexibility during S glycoprotein synthesis or transport [69], and avoid formation of unstable spike conformers. Although the spike’s temperature dependency may seem quite subtle, its relevance becomes clear from our comparison of the two SARS-2-S variants. Compared to the S^D614^ variant, the S^G614^ (pseudo)virus exhibited higher stability and slower loss of infectivity; this was evident at 37°C but not at 33°C. This agrees with the finding that two clinical isolates bearing either S^G614^ or S^D614^ exhibited comparable replication in human bronchial epithelial cells at 33°C, however the S^G614^ variant reached higher infectious virus titers at 37°C and 39°C [17]. Whether a similar temperature effect may also apply to other recently emerged SARS-CoV-2 spike variants, warrants further investigation.

Secondly, we demonstrated which of the 18 human TTSPs can activate SARS-2-S for virus entry. Many of these TTSPs are present in human respiratory tissue [13], however it is possible that the levels that we generated by ectopic expression may be above physiological levels. We confirmed that TMPRSS2 is an efficient and broad CoV spike activator. HAT proved effective on 229E-S and MERS-S, however it was less active on SARS-S and SARS-2-S, as reported earlier [33,34,58,63]. We did not see the recently reported activation of SARS-2-S by DESC1 [33,34] and TMPRSS11F [33], which belong to the same subfamily as HAT. This suggests that the level of TTSP activation depends on assay conditions, in particular the level of protease that is generated by plasmid transfection. Still, the most intriguing result regards TMPRSS13, which we and others [33,34,58] established as a second potent activator of SARS-S, SARS-2-S and MERS-S, but not 229E-S. Since this protease prefers cleavage sites with a second basic residue at positions P2 or P4 [70], it plausibly recognizes the S2′ site at the KR motif of SARS-S and SARS-2-S, and the RSAR motif of MERS-S. Such a motif is missing in the predicted S2′ sites of all common cold CoVs [35]. This raises the hypothesis that TMPRSS13 cleavability might be a CoV virulence factor, in analogy with the observation that this protease activates the hemagglutinin of some highly pathogenic avian influenza A viruses [68]. TMPRSS13 is highly expressed in different cell types of the human respiratory tract [33,71] and is also present in Calu-3 cells [13]. At least in these cells, SARS-CoV-2 replication relies on TMPRSS2 and, to a lesser extent, TMPRSS13, as evident from our knockdown experiments. It is important to note that SARS-2-S cleavability by other TTSPs than TMPRSS2 does not compromise clinical evaluation of camostat against COVID-19 (ClinicalTrials.gov identifiers: NCT04455815, NCT04321096, NCT04353284, NCT04355052, NCT04374019), since this molecule is a broad inhibitor of serine proteases including all TTSPs [33,72].

Thirdly, our data with SARS-2-S loop mutants show that the extended loop length and furin motif are equally important to achieve S1/S2 processing. Besides furin (or related proprotein convertases), also cathepsin B/L in the secretory pathway [73] might possibly perform S1/S2 pre-cleavage during S protein trafficking or during viral egress via a recently discovered lysosome-exocytic pathway [74]. All three enzymes were shown to cleave the S1/S2 sequence in an enzymatic assay [41]. Also, the possibility that other proteases besides furin can process the S1/S2 site, is supported the observation that furin knockout does not fully prevent cleavage of the S1/S2 site in SARS-2-S [42]. When unprimed, SARS-2-S pseudoviruses are strongly boosted towards the cathepsin B/L route. This likely explains the replication advantage of loop-deletion SARS-CoV-2 mutants in cathepsin L-rich Vero E6 cells [45–52]. The non-covalently linked S1/S2 form is less stable and a plausible disadvantage for endosomal entry. During virus traffic from the cell membrane to late endosomes (which takes up to 1 h [75]), S1 and S2 must remain associated under gradually more acidic conditions. The S1/S2 loop mutant (= unprimed) virions circumvent this problem, since they are only cleaved by cathepsins after reaching acidic endosomes. The superior stability of these mutants is evident from our thermostability experiments. In the case of wild-type SARS-2-S, pseudovirions shed by producer cells contained S1/S2 pre-cleaved spikes, which likely facilitates S2′ activation by cell surface proteases. Such a two-step cleavage process was demonstrated for MERS-S [43] and might also apply to SARS-2-S. On the other hand, its peculiar S1/S2 cleavage loop might broaden cleavability and allow that both (i.e. S1/S2 and S2′) cleavage events are performed during virus entry, by cell surface (like TTSP) proteases. Clearly, dedicated studies are still needed to fully understand the sequence and versatility of SARS-CoV-2 spike processing, and its role in determining the cell tropism of this virus.

Strikingly, also the more stable S^G614^ variant (which does undergo efficient S1/S2 priming) seemed more efficient in entering via the cathepsin route. The lower stability of S^D614^ was attributed to an unfavorable interprotomer contact that is not present in S^G614^ [22,23]. Besides, the superior cell entry of S^G614^ pseudovirus, seen in this and several other studies [15,19–23], was rationalized by structural evidence that the D614G substitution leads to a more open receptor binding conformation [22]. However, we found that the entry advantage of the S^G614^ variant was more pronounced in Vero E6 cells than in Calu-3 cells. Also our virus inhibition experiments with camostat, using cathepsin L-expressing Calu-3 cells, demonstrated that the more stable S^G614^ variant is more effective at using also the cathepsin entry route, which may broaden its cell tropism. Cathepsin-mediated CoV entry may thus have higher *in vivo* relevance than often assumed. Both cathepsin B and cathepsin L are present in human nasal tissue and lung tissue, being expressed in several types of airway epithelial cells [76].

Improved stability likely also explains why SARS-CoV-2-S^D614^ was superseded by the S^G614^ variant within a few months of circulation in humans [15]. Thanks to higher stability, the S^G614^ variant should have higher particle infectivity when shed, hence a smaller inoculum may be required to establish a new infection. This may explain why this variant not only shows higher [17], but also earlier [19] transmissibility in animal models. This is reminiscent of influenza virus, for which the link between hemagglutinin stability and transmissibility is well established [13,57]. Along the same line, we briefly addressed the spike’s pH stability, and observed that SARS-2-S, MERS-S and 229E-S are more stable at pH 6.3, the average pH of the nasal cavity, compared to pH 7.5. For SARS-S, this slightly acidic pH seemed to have a negative effect. This might point to a mechanism of adaptation to the pH of the upper or lower airways, with some analogy to influenza virus [13,57].

To conclude, we revealed mechanisms whereby the coronavirus spike protein is adjusted to match the temperature and protease conditions of the human airways. This insight will help to better comprehend coronavirus-host interaction and adaptation, and, in short term, will be highly valuable to understand the behavior of emerging spike mutants of SARS-CoV-2.

## Materials and methods

### Ethics statement

Samples of healthy lung tissue and healthy nasal tissue, each from three adult human donors, were obtained under the approval of the ethical committee from the University Hospital Leuven (UZ Leuven Biobanking S51577 and S59865). Written informed consent was obtained for lung tissue samples. Nasal tissue samples required no written consent, since this was secondary use of residual material from patients undergoing functional surgery.

### Cells, media and compounds

Unless stated otherwise, all cell incubations were done at 37°C. 16HBE [a gift from P. Hoet (Leuven, Belgium)] and Calu-3 (ATCC HTB-55) cells were grown in minimum essential medium (MEM) supplemented with 10% fetal calf serum (FCS), 0.1 mM non-essential amino acids (NEAA), 2 mM L-glutamine, and 10 mM HEPES. HEK293T cells (Thermo Fisher Scientific HCL4517), Vero E6 (ATCC CRL-1586), Huh-7 and HEL299 (human embryonic lung; ATCC CCL-137) cells were grown in Dulbecco’s modified Eagle’s medium (DMEM) supplemented with 10% FCS, 1 mM sodium pyruvate, 0.075% sodium bicarbonate and 0.1 mM NEAA. BHK-21J (baby hamster kidney fibroblast) cells [77] were kindly provided by P. Bredenbeek (Leiden, The Netherlands) and maintained in MEM supplemented with 10% FCS, 2 mM L-glutamine and 1% sodium bicarbonate.

The transduction medium consisted of DMEM supplemented with 2% FCS, 0.1 mM NEAA, 1 mM sodium pyruvate, 0.075% sodium bicarbonate or 10 mM HEPES, 100 U/mL of penicillin and 0.1 mg/mL of streptomycin. For virus infection experiments, the infection medium consisted of MEM supplemented with 0.1 mM NEAA, 2 mM L-glutamine, 10 mM HEPES, 100 U/mL of penicillin, 0.1 mg/mL of streptomycin and (for VeroE6 and Huh-7 cells) 2% FCS or (for Calu-3 cells) 0.2% FCS and 0.3% BSA.

Calu-3 cells stably overexpressing cathepsin L (Calu-3-CTSL) and control Calu-3 cells (Calu-3-EMPTY) were generated by retroviral transduction [78]. In brief, murine leukemia virus (MLV)-based transduction vectors containing pQCXIP-CTSL-cMYC or empty pQCXIP vector were generated by cotransfection of HEK293T cells with expression plasmids for MLV-gag/pol and VSV-G, and either pQCXIP-CTSL-cMYC or pQCXIP-EMPTY vector. At 18 h post transfection, the medium was exchanged and cells were incubated for an additional 48 h, before the supernatant was collected, centrifuged to remove cellular debris (2,000 x g, 10 min, room temperature) and filtered through a syringe filter with a pore size of 0.45 μm. Next, Calu-3 cells that were grown to ~25% confluency in T-25 flasks were transduced for 48 h with a 1:5 (v/v) dilution of the transduction particles, before the medium was exchanged and cells were further incubated in the presence of 4 μg/ml puromycin. Once the selection process was finished (as indicated by death of non-transduced Calu-3 cells that were also incubated in the presence of 4 μg/ml puromycin), the puromycin concentration was reduced to 0.5 μg/ml for subculturing of the cells.

Camostat mesylate was purchased from Sigma-Aldrich, whereas GS-441524 was from Carbosynth. E64d and chloromethylketone (dec-RVKR-CMK) were purchased from Enzo Life Sciences.

### Plasmids

For 16 out of 18 human TTSPs and for furin, we used expression plasmids bearing a C-terminal flag tag, that we purchased from GenScript. As demonstrated earlier [13], these plasmids yielded similar protein levels for the various TTSPs. For HAT and DESC1, the expression plasmids were validated in another study [58]. To express the CoV receptors, we used plasmids encoding human ACE2 [79]; human DPP4 [80] and human APN [63].

The plasmids to express C-terminal V5-tagged SARS-S and MERS-S [protein sequences from clinical virus isolates: NCBI accession numbers AAP33697.1 and YP_009047204.1 (reference sequence), respectively] were already reported [81,82]. To create the SARS-2-S-V5 expression plasmid, we used a starting plasmid carrying a codon-optimized full-length SARS-2-S coding sequence (reference sequence, early pandemic D614 variant; NCBI accession number YP_009724390.1) that was generously provided by K. Dallmeier (Leuven, Belgium) [83]. A C-terminal V5-tag was added and the construct was subcloned into the pCAGGS vector using the NEBuilder HiFi DNA Assembly kit (New England Biolabs). Likewise, a V5 tag was introduced into a pCAGGS-based plasmid encoding 229E-S [protein sequence identical to NCBI accession number NP_073551.1 (reference sequence)] [84]. Mutations in the S coding sequence were introduced via PCR with overlapping primers, and inserted into pCAGGS using the NEBuilder HiFi DNA Assembly kit. All plasmids were subjected to sequencing analysis to verify the presence of the desired mutations and absence of any unwanted mutations.

### Analysis of protease or receptor expression

To assess expression of the proteases, cells were lysed in RIPA buffer supplemented with protease inhibitor cocktail (both from Thermo Fisher Scientific). Tissue samples were homogenized using 2.8 mm zirconium oxide beads (Precellys24, Bertin Technologies). Homogenates were cleared by centrifugation (5 min, 9,000 g). The lysates were boiled for 5 min at 95°C in 1x XT sample buffer containing 1x XT reducing agent (both from Bio-Rad) and resolved on 4–12% Bis-Tris XT precast gels (Bio-Rad). The proteins were transferred to polyvinylidene difluoride membranes (Bio-Rad), blocked with 5% low-fat milk solution, and probed for 1 h with primary antibody followed by 45 min with secondary antibody. Bands were detected using SuperSignal West Femto Maximum Sensitivity Substrate (Thermo Fisher Scientific) and a ChemiDoc XRS+ system (Bio-Rad) [see S4 Table for a list of all antibodies].

For analysis of coronavirus receptor expression, total RNA was extracted using a ReliaPrep RNA Cell Miniprep System (Promega), and 0.5 μg of RNA was converted to cDNA with a high-capacity cDNA reverse transcription kit (Thermo Fisher Scientific). BRYT Green dye-based quantitative PCR (qPCR) was performed with GoTaq qPCR Master Mix (Promega) and intron-spanning primer pairs in an ABI 7500 Fast real-time PCR system (Applied Biosciences) (see primer sequences in S4 Fig). Expression data were normalized to housekeeping gene *ACTB*.

### Production of S-pseudotyped viruses and transduction experiments

The method to produce firefly luciferase (fLuc)-expressing MLV pseudovirus carrying CoV S-protein, was previously described [85]. In brief, HEK293T cells seeded in 6-well plates, were transfected using Lipofectamine 2000 (Life Technologies), with a mixture of plasmids encoding MLV gag-pol, the fLuc reporter and V5-tagged S-protein. At 4 h post transfection, the medium was replaced by medium with 2% FCS. Pseudoparticle production was done at 33°C or 37°C, as specified in the Figure legends. At 48 h, the pseudovirus-containing supernatants were harvested, clarified by centrifugation and stored at -80°C.

For MLV pseudovirus transduction (always performed at 37°C), Calu-3 or Vero E6 cells were seeded in white 96-well plates and one day later exposed to 100 μl virus stock. In the case of HEK293T, the cells were first transfected with the receptor- and TTSP- expression plasmids, at 24 h before transduction. In some experiments, protease inhibitors, i.e. camostat mesylate, E64d, chloromethylketone (all at 50 μM) or 1% DMSO (solvent control), were added at 2 h before pseudovirus transduction. At 6 h after transduction, pseudovirus and compounds were removed and fresh medium was added. Three days later, fLuc activity was measured using a luciferase assay system kit and GloMax Navigator Microplate Luminometer (both from Promega).

To generate S-pseudotyped vesicular stomatitis virus (VSV) particles, BHK-21J cells were transfected with the respective S protein expression plasmids, and one day later infected (MOI = 2) with GFP-encoding VSVΔG backbone virus (purchased from Kerafast) [83]. Two hours later, the medium was replaced by medium containing anti-VSV-G antibody (I1-hybridoma, ATCC CRL-2700) to neutralize residual VSV-G input. After 24 h incubation at 32°C or 37°C, the supernatants were harvested, and used to transduce Huh-7 (for MERS-S-bearing VSV particles); Vero E6 (for SARS-S and SARS-2-S); or 16HBE cells (for 229E-S). After 18 h (Huh-7 and Vero E6 cells) or 24 h (16HBE cells) incubation at 37°C, the cells were fixed with 4% paraformaldehyde. After staining with DAPI, the percentage GFP-positive cells was quantified on a CellInsight CX5/7 High Content Screening platform (Thermo Fisher Scientific) with Thermo Fisher Scientific HCS Studio (v.6.6.0) software.

To assess thermostability of the pseudoparticles, they were incubated for 1 h in tubes, at a temperature of 33, 35, 37, 39 or 41°C, or at 4°C included as control. They were then transduced into receptor- and TMPRSS2-transfected HEK293T cells. Two hours later, particles were removed and fresh medium was added. To assess pH stability, the pseudoparticle stocks were adjusted to pH 6.3, 7.5 or 8.0, by addition of phosphate buffer (pH 5.9), or HEPES buffer (pH 7.7 and 8.2). After 1 h incubation at 4°C, the stocks were diluted in HEPES-buffered transduction medium and transduced into receptor- and TMPRSS2-transfected HEK293T cells as described above.

### Assessment of HCoV-229E virus replication

To assess the effect of temperature on the replication efficiency of HCoV-229E, HEL299 cells were infected with HCoV-229E at a multiplicity of infection (MOI) of 100 x 50% cell culture infective dose (CCID_50_) per well (determined at 37°C), and incubated at 33°C, 35°C, 37°C or 39°C. The number of viral genome copies in the supernatant was determined at 0, 1, 2, 3 and 4 days post infection (p.i.), using RT-qPCR with HCoV-229E N-gene specific primers and probe, as described elsewhere [86]. In parallel, titers of infectious virus were determined at the four different temperatures by end-point dilution, and calculated by the CCID_50_ method of Reed and Muench [87].

### Assessment of SARS-CoV-2 virus replication

Two SARS-CoV-2 strains bearing residue S^D614^ or S^G614^ were recovered from nasopharyngeal swabs of RT-qPCR-confirmed asymptomatic human cases. To prepare virus stocks, the isolates underwent two passages on Huh-7 cells. Full genome sequencing using MinION (Oxford Nanopore Technologies) confirmed the presence of residue D or G at spike position 614. The S^D614^ strain [SARS-CoV-2/Belgium/GHB-03021/2020 (GISAID accession number EPI_ISL_407976)] belongs to clade 19B / A, while the S^G614^ strain (GISAID accession number EPI_ISL_888706) belongs to clade 20A.EU2 / B.1.160.

To determine replication efficiency, the two SARS-CoV-2 strains were added to Calu-3 cells at an MOI of 100 CCID_50_ per well, as determined by end-point dilution titration on Calu-3 cells at 37°C. At different time points after incubation at 33°C or 37°C, the supernatants were collected and frozen at -80°C. The number of viral genome copies was determined by RT-qPCR using the CellsDirect One-Step RT-qPCR kit (Invitrogen), as described before [86], and the US CDC 2019-nCoV_N1 primer-probe set (IDT) [88]. The 2019-nCoV_N Positive Control plasmid (IDT) was used as a plasmid standard. In parallel, titers of infectious virus in these supernatants were determined by end-point dilution on Vero E6 cells, and calculated by the CCID_50_ method of Reed and Muench [87].

Inhibition of virus replication by camostat, E64d or GS-441524 was determined by adding the compounds to Calu-3-CTSL or Calu-3-EMPTY cells, which were infected with virus (MOI: 100 CCID_50_ per well) two hours later. After 3 days incubation at 37°C, the cells were immunostained for viral dsRNA, using the J2 dsRNA antibody (see S4 Table), combined with nuclear Hoechst staining [89]. The percentage green cells was quantified using a CellInsight CX5 High Content Screening platform (Thermo Fisher Scientific).

### Western blot analysis of S protein expression

To analyze S protein expression in HEK293T cells, the plasmids encoding V5-tagged S protein were transfected into these cells, using Lipofectamine 2000. Four hours later, the medium was replaced by medium with 2% FCS and the cells were incubated for another 48 h. Cells were washed once with PBS and lysed in RIPA buffer supplemented with protease inhibitor cocktail (both from Thermo Fisher Scientific).

For analysis of S protein incorporation into pseudoparticles, a volume of 600 μl of S-pseudotyped MLV virus was loaded onto a 20% (w/v) sucrose cushion (volume 50 μl) and subjected to high-speed centrifugation (25,000 g for 120 min at 4°C). Thereafter, 630 μl of supernatant was removed and the residual volume was mixed with 30 μl loading dye mastermix, consisting of RIPA buffer supplemented with protease inhibitor cocktail (both from Thermo Fisher Scientific) and 1x XT sample buffer containing 1x XT reducing agent (both from Bio-Rad). The samples were heated for 5 min at 95°C and subjected to SDS-PAGE and immunoblotting, as above. Antibody details can be found in S4 Table.

### siRNA-mediated protease knockdown

All experimental details for siRNA-mediated knockdown of TMPRSS2 and TMPRSS13 in Calu-3 cells can be found elsewhere [13]. A scrambled siRNA was included for control. Briefly, cells seeded in black 96-well plates were transfected with 10 nM siRNA using Lipofectamine RNAiMAX, and after 24 h incubation at 37°C, the transfection medium was replaced by Calu-3 infection medium. One day later, the cells were infected with SARS-CoV-2 virus at an MOI of 100 CCID_50_. At 72 h p.i., cells were immunostained for viral dsRNA and submitted to high-content imaging, as explained above.

### Data and statistical analysis

All numerical values that were used to generate graphs are available in the Supporting Information (S1 Data). Statistical analysis was performed using GraphPad Prism (version 8.4.3). Statistical significance of differences between datasets was analyzed by ordinary one-way ANOVA with Dunnett’s correction; Fisher’s LSD test; or unpaired two-tailed t-test, as stated in the Figure legends. P ≤ 0.05 was considered significant. Statistical significance is reported as: *, P ≤ 0.05; **, P ≤ 0.01; ***, P ≤ 0.001; ****, P ≤ 0.0001.

## Supporting information

S1 Table Deletions in the S1/S2 cleavage loop, observed in the GISAID database. (PDF)

S2 Table Substitutions in the S1/S2 cleavage loop, observed in the GISAID database. (PDF)

S3 Table Deletions and substitutions in the S1/S2 cleavage loop, observed after passaging in cell culture. (PDF)

S4 Table Antibodies for western blot detection and immunostaining. (PDF)

S1 Fig HCoV-229E shows temperature-dependent replication with a preference for 33°C and 35°C. (A) HEL299 cells were infected with HCoV-229E and incubated at 33°C, 35°C, 37°C or 39°C. At different time points p.i., supernatants were collected to determine the viral genome copy number, using RT-qPCR. Values are the mean of three experiments, performed in triplicate. (B) Titers of infectious virus were determined at day 5 p.i., by the CCID_50_ end-point dilution method (N = 3). ns, P > 0.05; *, P ≤ 0.05; **, P ≤ 0.01; ****, P ≤ 0.0001 (Fisher’s LSD test; versus 33°C condition).
(TIF)

S2 Fig Pseudovirus entry is similar at 33°C and 37°C. S-pseudotyped MLV particles were produced at their optimal production temperature (33°C or 37°C), harvested and used for transduction of HEK293T target cells expressing the appropriate receptor and TMPRSS2. Transduction was carried out for 2 h at either 33°C or 37°C, after which particles were removed, fresh medium was added and further incubation was done at 33°C. At day 3 post transduction, particle entry was measured by luminescence read-out (mean ± SEM from three experiments). ns, P > 0.05 (two-tailed unpaired t-test, 37°C versus 33°C).
(TIF)

S3 Fig Effect of production temperature on infectivity of S-pseudotyped VSV particles. GFP-encoding pseudoviruses bearing different S-proteins were produced in BHK-21J cells at either 32 or 37°C. Next, they were transduced into target cells, i.e. Vero E6 for SARS-S- and SARS-2-S-bearing pseudoviruses; Huh-7 for MERS-S; or 16HBE for 229E-S, and incubated at 37°C. One day later, the number of GFP-expressing cells was quantified by high-content imaging. N = 2, performed in triplicate. **, P ≤ 0.01; ***, P ≤ 0.001; ****, P ≤ 0.0001 (two-tailed unpaired t-test; 37°C versus 32°C).
(TIF)

S4 Fig Expression of coronavirus receptors in Calu-3 and Vero E6 cells, and in human lung tissue and human nasal tissue. The heatmap shows mRNA levels of the receptor transcripts (relative to β-actin), determined by RT-qPCR. The Table shows the primer sequences used for RT-qPCR analysis.
(TIF)

S5 Fig Exogenous trypsin restores the entry defect in Calu-3 cells of S1/S2 loop mutant SARS-2-S pseudoviruses. The pseudoparticles were allowed to bind to Calu-3 cells for 1 h at 4°C, after which unbound particles were removed and DMEM with 10 μg/ml TPCK-trypsin was added. After 2 h at 37°C, the medium was replaced by Calu-3 growth medium. Results are the mean ± SEM; N = 3. ***, P ≤ 0.001 (two-tailed unpaired t-test; trypsin-treated versus -untreated condition).
(TIF)

S1 Data Numerical values used to generate graphs. (XLSX)
