# Supplementary material for: The SARS-CoV-2 and other human coronavirus spike proteins are fine-tuned towards temperature and proteases of the human airways
Source: PLoS Pathog. 2021 Apr 22;17(4):e1009500. doi: 10.1371/journal.ppat.1009500 (PMC8061995; doi:10.1371/journal.ppat.1009500)
Supplement: S1 Table — (PDF) [file ppat.1009500.s001.pdf]

Supplementary Table S1. Deletions in the S1/S2 cleavage loop, observed in the GISAID database.

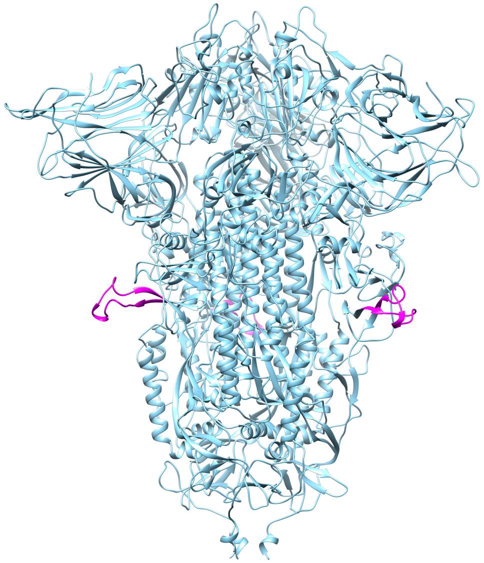

Sequence	Isolate	Accession ID	Passage details
SYQTQTNSP**RR**A**R**SVASQSII	hCoV-19/Wuhan/WIV04/2019	EPI_ISL_402124	Original isolate - reference
SYQTQTNSP**RR**A**R**-IASQSII	hCoV-19/Russia/Moscow-PMVL-12/2020	EPI_ISL_572398	Vero E6 passaged
SYQTQT--------IASQSII	hCoV-19/England/20238034404/2020	EPI_ISL_471513	Original isolate
	hCoV-19/England/2/2020	EPI_ISL_610245	Vero E6 passaged
SYQTQT----------SQSII	hCoV-19/Hong Kong/XM-PII-S4/2020	EPI_ISL_417443	Vero E6 passaged [1]
	hCoV-19/Slovenia/4265/2020	EPI_ISL_635205	Original isolate
	hCoV-19/Ireland/D-NVRL-AIIDV1469v1/2020	EPI_ISL_791294	Original isolate
	hCoV-19/France/PAC-IHU-3386-1/2020	EPI_ISL_1109982	Original isolate
SYQTQ------------QSII	hCoV-19/USA/MO-WUSTL069/2020	EPI_ISL_493086	Original isolate
SY-----SP**RR**A**R**SVASQSII	hCoV-19/Malaysia/IMR_WC1098/2020	EPI_ISL_430442	Vero E6 passaged
	hCoV-19/Taiwan/CGMH-CGU-22/2020	EPI_ISL_444275	Vero E6 passaged
	hCoV-19/France/IHU-0914/2020	EPI_ISL_568990	Original isolate
	hCoV-19/France/IHU-0921/2020	EPI_ISL_568991	Original isolate
	hCoV-19/England/NOTT-11974A/2020	EPI_ISL_627304	Original isolate

	hCoV-19/France/PAC-MEPHI-1208/2020	EPI_ISL_644402	Original isolate
	hCoV-19/Malaysia/IMR-WI124/2020	EPI_ISL_718278	Original isolate
	hCoV-19/England/QEUH-BA174D/2020	EPI_ISL_719718	Original isolate
	hCoV-19/France/OCC-IHUCOVID-1648/2020	EPI_ISL_860094	Vero E6 passaged
	hCoV-19/Russia/Moscow_PMVL-13/2020	EPI_ISL_872628	Vero E6 passaged
	hCoV-19/Russia/Moscow_PMVL-14/2020	EPI_ISL_872629	Vero E6 passaged
	hCoV-19/Russia/Moscow_PMVL-15/2020	EPI_ISL_872630	Vero E6 passaged
	hCoV-19/Russia/Moscow_PMVL-16/2020	EPI_ISL_872631	Vero E6 passaged
	hCoV-19/Russia/Moscow_PMVL-18/2020	EPI_ISL_872633	Vero E6 passaged
	hCoV-19/Russia/Moscow_PMVL-20/2020	EPI_ISL_872634	Vero E6 passaged
	hCoV-19/France/PAC-IHUCOVID-0924/2020	EPI_ISL_900088	Original isolate
	hCoV-19/France/PAC-IHUCOVID-0920/2020	EPI_ISL_900094	Original isolate
	hCoV-19/France/PAC-IHU-2456/2020	EPI_ISL_982228	Original isolate
	hCoV-19/France/PAC-IHU-3113i/2020	EPI_ISL_982229	Vero E6 passaged
	hCoV-19/France/PAC-IHU-3126i/2020	EPI_ISL_982230	Vero E6 passaged
	hCoV-19/Italy/CAM-TIGEM-2053/2021	EPI_ISL_1056669	Original isolate
	hCoV-19/France/PAC-IHU-3415-1/2020	EPI_ISL_1110006	Original isolate
SY---TNSP**RR**A**R**SVASQSII	hCoV-19/England/ALDP-C3C870/2020	EPI_ISL_731669	Original isolate

All 725,325 available spike sequences were downloaded from the Global Initiative on Sharing All Influenza Data (GISAID) database on 12/03/2021 [2]. The Table shows deletions in the S1/S2 cleavage loop, indicated in magenta in the trimeric spike protein (Figure on top; based on PDB 6ZGE [3], in which we modelled the cleavage loop with SWISS-MODEL [4]).

1. Lau SY, Wang P, Mok BW, Zhang AJ, Chu H, Lee AC, et al. Attenuated SARS-CoV-2 variants with deletions at the S1/S2 junction. Emerg Microbes Infect. 2020;9(1):837-842. doi: 10.1080/22221751.2020.1756700. PubMed PMID: 32301390.
2. Shu Y, McCauley J. GISAID: Global initiative on sharing all influenza data - from vision to reality. Euro Surveill. 2017;22(13). doi: 10.2807/1560-7917.Es.2017.22.13.30494. PubMed PMID: 28382917.
3. Wrobel AG, Benton DJ, Xu P, Roustan C, Martin SR, Rosenthal PB, et al. SARS-CoV-2 and bat RaTG13 spike glycoprotein structures inform on virus evolution and furin-cleavage effects. Nat Struct Mol Biol. 2020;27(8):763-767. doi: 10.1038/s41594-020-0468-7. PubMed PMID: 32647346.
4. Waterhouse A, Bertoni M, Bienert S, Studer G, Tauriello G, Gumienny R, et al. SWISS-MODEL: homology modelling of protein structures and complexes. Nucleic Acids Res. 2018;46(W1):W296-W303. doi: 10.1093/nar/gky427. PubMed PMID: 29788355.
