# Supplementary material for: The SARS-CoV-2 and other human coronavirus spike proteins are fine-tuned towards temperature and proteases of the human airways
Source: PLoS Pathog. 2021 Apr 22;17(4):e1009500. doi: 10.1371/journal.ppat.1009500 (PMC8061995; doi:10.1371/journal.ppat.1009500)
Supplement: S2 Table — (PDF) [file ppat.1009500.s002.pdf]

Supplementary Table S2. Substitutions in the S1/S2 cleavage loop, observed in the GISAID database.

Sequence	Total number of sequences (/725,325)	% unpassaged viruses
SYQTQTNSP**RRAR**SVASQSII	533,680	99.5
TYQTQTNSP**RRAR**SVASQSII	522	99.8
GYQTQTNSP**RRAR**SVASQSII	56	100
**R**YQTQTNSP**RRAR**SVASQSII	8	100
IYQTQTNSP**RRAR**SVASQSII	6	100
SFQTQTNSP**RRAR**SVASQSII	79	100
SYHTQTNSP**RRAR**SVASQSII	4970	99.5
SY**R**TQTNSP**RRAR**SVASQSII	980	97.5
SY**K**TQTNSP**RRAR**SVASQSII	16	100
SYPTQTNSP**RRAR**SVASQSII	8	100
SYLTQTNSP**RRAR**SVASQSII	3	100
SYQIQTNSP**RRAR**SVASQSII	171	100
SYQAQTNSP**RRAR**SVASQSII	22	100
SYQSQTNSP**RRAR**SVASQSII	11	100
SYQPQTNSP**RRAR**SVASQSII	6	100
SYQNQTNSP**RRAR**SVASQSII	4	100
SYQTHTNSP**RRAR**SVASQSII	7870	99.7
SYQTPTNSP**RRAR**SVASQSII	1568	100
SYQT**R**TNSP**RRAR**SVASQSII	157	100
SYQT**K**TNSP**RRAR**SVASQSII	8	100
SYQTLTNSP**RRAR**SVASQSII	6	100
SYQTQINSP**RRAR**SVASQSII	197	99.5
SYQTQPNSP**RRAR**SVASQSII	11	100
SYQTQANSP**RRAR**SVASQSII	7	100
SYQTQNNSP**RRAR**SVASQSII	2	100
SYQTQSNSP**RRAR**SVASQSII	1	100
SYQTQT**K**SP**RRAR**SVASQSII	539	99.8
SYQTQTSSP**RRAR**SVASQSII	36	100
SYQTQTYSP**RRAR**SVASQSII	6	100
SYQTQTTSP**RRAR**SVASQSII	6	100
SYQTQTHSP**RRAR**SVASQSII	5	100
SYQTQTISP**RRAR**SVASQSII	3	100
SYQTQTDSP**RRAR**SVASQSII	2	100
SYQTQTNFP**RRAR**SVASQSII	25	100
SYQTQTNTP**RRAR**SVASQSII	7	100
SYQTQTNYP**RRAR**SVASQSII	2	100
SYQTQTNAP**RRAR**SVASQSII	1	100
SYQTQTNSH**RRAR**SVASQSII	153,573	99.7
SYQTQTNS**RRRAR**SVASQSII	906	99.4
SYQTQTNSL**RRAR**SVASQSII	262	100
SYQTQTNSS**RRAR**SVASQSII	44	95.5
SYQTQTNST**RRAR**SVASQSII	1	100
SYQTQTNSPW**RAR**SVASQSII	14	85.7
SYQTQTNSPQ**RAR**SVASQSII	9	55.6
SYQTQTNSPL**RAR**SVASQSII	4	50
SYQTQTNSP**R**W**AR**SVASQSII	19	100
SYQTQTNSP**R**L**AR**SVASQSII	14	100

SYQTQTNSPRQARSVASQSII	9	100
SYQTQTNSPRPARSVASQSII	1	100
SYQTQTNSPRRVRSVASQSII	264	100
SYQTQTNSPRRTRSVASQSII	29	100
SYQTQTNSPRRSRSVASQSII	14	92.9
SYQTQTNSPRRPRSVASQSII	1	100
SYQTQTNSPRRAGSVASQSII	2	100
SYQTQTNSPRRARGVASQSII	3	66.7
SYQTQTNSPRRARRVASQSII	1	100
SYQTQTNSPRRARSLASQSII	325	100
SYQTQTNSPRRARSIASQSII	80	100
SYQTQTNSPRRARSVVSQSII	1901	99.7
SYQTQTNSPRRARSVSSQSII	180	98.9
SYQTQTNSPRRARSVTSQSII	16	100
SYQTQTNSPRRARSVDSQSII	1	100
SYQTQTNSPRRARSVAIQSII	284	99.6
SYQTQTNSPRRARSVARQSII	95	96.8
SYQTQTNSPRRARSVAGQSII	5	100
SYQTQTNSPRRARSVAKQSII	4	100
SYQTQTNSPRRARSVANQSII	2	100
SYQTQTNSPRRARSVASRSII	119	99.1
SYQTQTNSPRRARSVASHSII	60	100
SYQTQTNSPRRARSVASLSII	12	100
SYQTQTNSPRRARSVASKSII	2	100
SYQTQTNSPRRARSVASQFII	109	100
SYQTQTNSPRRARSVASQAII	71	100
SYQTQTNSPRRARSVASQPII	18	94.4
SYQTQTNSPRRARSVASQYII	4	100
SYQTQTNSPRRARSVASQCII	2	100
SYQTQTNSPRRARSVASQSVI	13	100
SYQTQTNSPRRARSVASQSFI	9	100
SYQTQTNSPRRARSVASQSLI	1	100
SYQTQTNSPRRARSVASQSTI	1	100
SYQTQTNSPRRARSVASQSMI	1	100
SYQTQTNSPRRARSVASQSIV	5	100
SYQTQTNSPRRARSVASQSIF	2	100

All 725,325 available spike sequences were downloaded from the GISAID database on 12/03/2021 [1] and variation in the S1/S2 loop was analyzed. The % unpassaged viruses represents the % of sequences obtained from viruses that were not passaged in cell culture before sequence analysis. Only single variations are shown, combinations also occur.

1. Shu Y, McCauley J. GISAID: Global initiative on sharing all influenza data - from vision to reality. Euro Surveill. 2017;22(13). doi: 10.2807/1560-7917.Es.2017.22.13.30494. PubMed PMID: 28382917.
