# Supplementary material for: The SARS-CoV-2 and other human coronavirus spike proteins are fine-tuned towards temperature and proteases of the human airways
Source: PLoS Pathog. 2021 Apr 22;17(4):e1009500. doi: 10.1371/journal.ppat.1009500 (PMC8061995; doi:10.1371/journal.ppat.1009500)
Supplement: S3 Table — (PDF) [file ppat.1009500.s003.pdf]

Supplementary Table S3. Deletions and substitutions in the S1/S2 cleavage loop, observed after passaging in cell culture.

Sequence	Report
SYQTQTNSP**RR**A**R**SVASQSII	Original isolate - reference
SY-----SP**RR**A**R**SVASQSII	Lau, et al. 2020 [1];<br>Ogando, et al. 2020 [2];<br>Liu, et al. 2020 [3]
SYQTQT-------SVASQSII	Lau, et al. 2020 [1];<br>Liu, et al. 2020 [3]
SYQTQT--------IASQSII	Davidson, et al. 2020 [4]
SYQTQT----------SQSII	Lau, et al. 2020 [1];<br>Ogando, et al. 2020 [2];<br>Sasaki, et al. 2020 [5];<br>Lamers er al. 2021 [6]
SYQTQTNS-**RR**A**R**SVASQSII	Klimstra, et al. 2020 [7]
SYQTQTNS-----SVASQSII	Klimstra, et al. 2020 [7]
SYQTQTNS---------QSII	Klimstra, et al. 2020 [7]
SYQTQTNSP**R**-------QSII	Sasaki, et al. 2020 [5];<br>Klimstra, et al. 2020 [7];<br>Zhu, et al. 2020 [8]
SYQTQTNSPQ-------QSII	Klimstra, et al. 2020 [7]
SYQTQTNSP**RR**A**R**SVA---II	Sasaki, et al. 2020 [5]
SYQTQTNSPLVGSSVASQSII	Klimstra, et al. 2020 [7]
SYQTQTNSPQ**R**A**R**SVASQSII	Klimstra, et al. 2020 [7]
SYQTQTNSPL**R**A**R**SVASQSII	Klimstra, et al. 2020 [7]<br>Lamers er al. 2021 [6]
SYQTQTNSP**R**QA**R**SVASQSII	Klimstra, et al. 2020 [7]
SYQTQTNSP**RR**AHSVASQSII	Sasaki, et al. 2020 [5]<br>Lamers er al. 2021 [6]
SYQTQTNSP**RR**ASSVASQSII	Klimstra, et al. 2020 [7]
SYQTQTNSP**RR**A**R**GVASQSII	Lamers er al. 2021 [6]

1. Lau SY, Wang P, Mok BW, Zhang AJ, Chu H, Lee AC, et al. Attenuated SARS-CoV-2 variants with deletions at the S1/S2 junction. Emerg Microbes Infect. 2020;9(1):837-842. doi: 10.1080/22221751.2020.1756700. PubMed PMID: 32301390.
2. Ogando NS, Dalebout TJ, Zevenhoven-Dobbe JC, Limpens R, van der Meer Y, Caly L, et al. SARS-coronavirus-2 replication in Vero E6 cells: replication kinetics, rapid adaptation and cytopathology. J Gen Virol. 2020;101(9):925-940. doi: 10.1099/jgv.0.001453. PubMed PMID: 32568027.
3. Liu Z, Zheng H, Lin H, Li M, Yuan R, Peng J, et al. Identification of common deletions in the spike protein of severe acute respiratory syndrome coronavirus 2. J Virol. 2020;94(17). doi: 10.1128/JVI.00790-20. PubMed PMID: 32571797.
4. Davidson AD, Williamson MK, Lewis S, Shoemark D, Carroll MW, Heesom KJ, et al. Characterisation of the transcriptome and proteome of SARS-CoV-2 reveals a cell passage induced in-frame deletion of the furin-like cleavage site from the spike glycoprotein. Genome Med. 2020;12(1):68. doi: 10.1186/s13073-020-00763-0. PubMed PMID: 32723359.
5. Sasaki M, Uemura K, Sato A, Toba S, Sanaki T, Maenaka K, et al. SARS-CoV-2 variants with mutations at the S1/S2 cleavage site are generated in vitro during propagation in TMPRSS2-deficient cells. PLoS Pathog. 2021;17(1):e1009233. doi: 10.1371/journal.ppat.1009233. PubMed PMID: 33476327.
6. Lamers MM, Mykytyn AZ, Breugem TI, Wang Y, Wu DC, Riesebosch S, et al. Human airway cells prevent SARS-CoV-2 multibasic cleavage site cell culture adaptation. bioRxiv [Preprint]. 2021 bioRxiv 2021.01.22.427802 [posted 2021 Jan 22; cited 2021 Mar 12]. Available from: https://www.biorxiv.org/content/10.1101/2021.01.22.427802v1 doi: 10.1101/2021.01.22.427802.
7. Klimstra WB, Tilston-Lunel NL, Nambulli S, Boslett J, McMillen CM, Gilliland T, et al. SARS-CoV-2 growth, furin-cleavage-site adaptation and neutralization using serum from acutely infected hospitalized COVID-19 patients. J Gen Virol. 2020;101(11):1156-1169. doi: 10.1099/jgv.0.001481. PubMed PMID: 32821033.
8. Zhu Y, Feng F, Hu G, Wang Y, Yu Y, Zhu Y, et al. A genome-wide CRISPR screen identifies host factors that regulate SARS-CoV-2 entry. Nat Commun. 2021;12(1):961. doi: 10.1038/s41467-021-21213-4. PubMed PMID: 33574281.
