# Supplementary material for: The SARS-CoV-2 and other human coronavirus spike proteins are fine-tuned towards temperature and proteases of the human airways
Source: PLoS Pathog. 2021 Apr 22;17(4):e1009500. doi: 10.1371/journal.ppat.1009500 (PMC8061995; doi:10.1371/journal.ppat.1009500)
Supplement: S4 Table — (PDF) [file ppat.1009500.s004.pdf]

Supplementary Table S4. Antibodies for western blot detection and immunostaining.

Antibody	Company<br>and catalog number	Host and clonality	Dilution
Anti-TMPRSS13	Sigma-Aldrich<br>SAB2700555	Rabbit polyclonal	1/1000
Anti-TMPRSS2	Thermo Fisher Scientific<br>PA5-14265	Rabbit polyclonal	1/1000
Anti-Cathepsin L	Thermo Fisher Scientific<br>BMS166	Mouse monoclonal	1/1000
Anti-Cathepsin B	Thermo Fisher Scientific<br>MA5-32651	Rabbit monoclonal	1/1000
Anti-MLV P30	Abcam<br>Ab130757	Mouse monoclonal	1/1000
Anti-V5 tag	Thermo Fisher Scientific<br>R960-25	Mouse monoclonal	1/1000
Anti-clathrin heavy chain	BD Biosciences<br>610499	Mouse monoclonal	1/1000
Anti-β actin	Sigma-Aldrich<br>A5441	Mouse monoclonal	1/8000
Anti-dsRNA	Scicons<br>J2	Mouse monoclonal	1/1000
Anti-mouse-<br>AlexaFluor488	Thermo Fisher Scientific<br>A-21131	Goat polyclonal	1/1000
Anti-rabbit-HRP	Dako<br>P0399	Swine polyclonal	1/4000 to<br>1/10000
Anti-mouse-HRP	Dako<br>P0447	Goat polyclonal	1/4000 to<br>1/10000
