# Supplementary figures and images for: The SARS-CoV-2 and other human coronavirus spike proteins are fine-tuned towards temperature and proteases of the human airways
Source: PLoS Pathog. 2021 Apr 22;17(4):e1009500. doi: 10.1371/journal.ppat.1009500 (PMC8061995; doi:10.1371/journal.ppat.1009500)

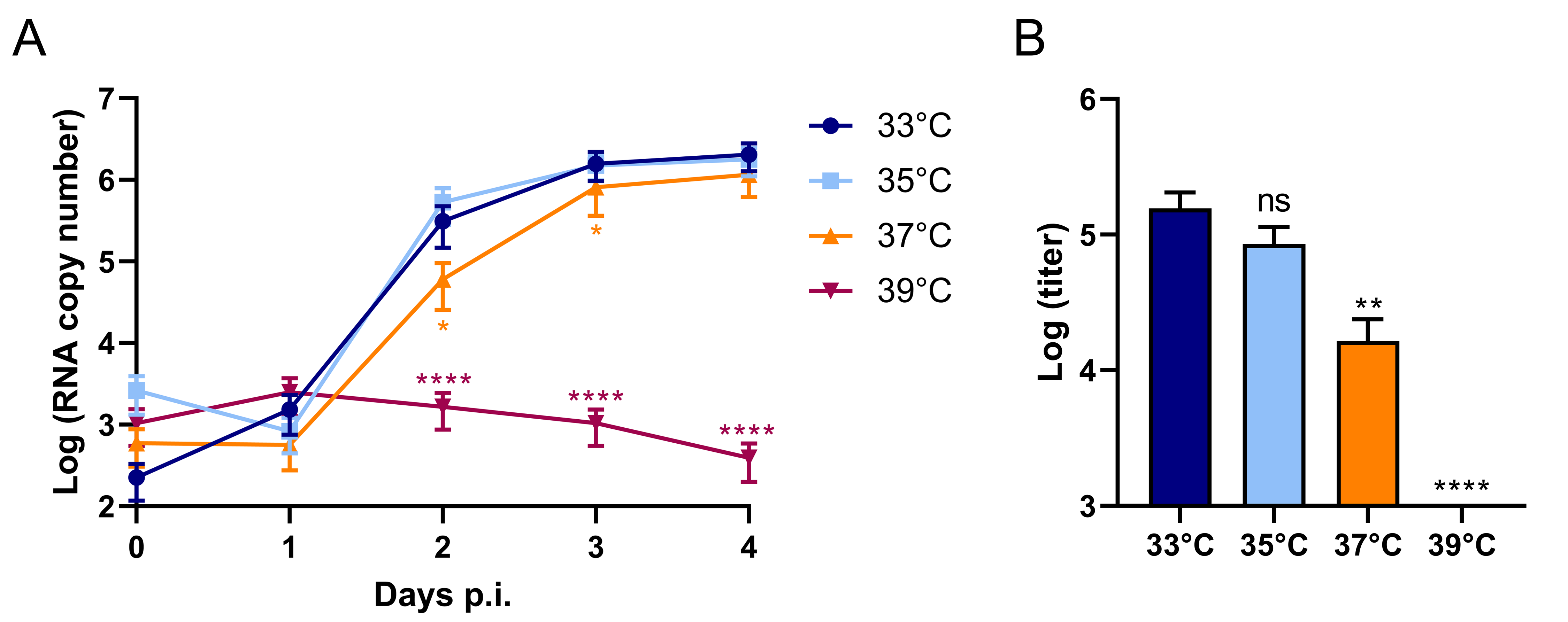

Supplement: S1 Fig — (A) HEL299 cells were infected with HCoV-229E and incubated at 33°C, 35°C, 37°C or 39°C. At different time points p.i., supernatants were collected to determine the viral genome copy number, using RT-qPCR. Values are the mean of three experiments, performed in triplicate. (B) Titers of infectious virus were determined at day 5 p.i., by the CCID50 end-point dilution method (N = 3). ns, P > 0.05; *, P ≤ 0.05; **, P ≤ 0.01; ****, P ≤ 0.0001 (Fisher’s LSD test; versus 33°C condition). (TIF) [file ppat.1009500.s005.tif]

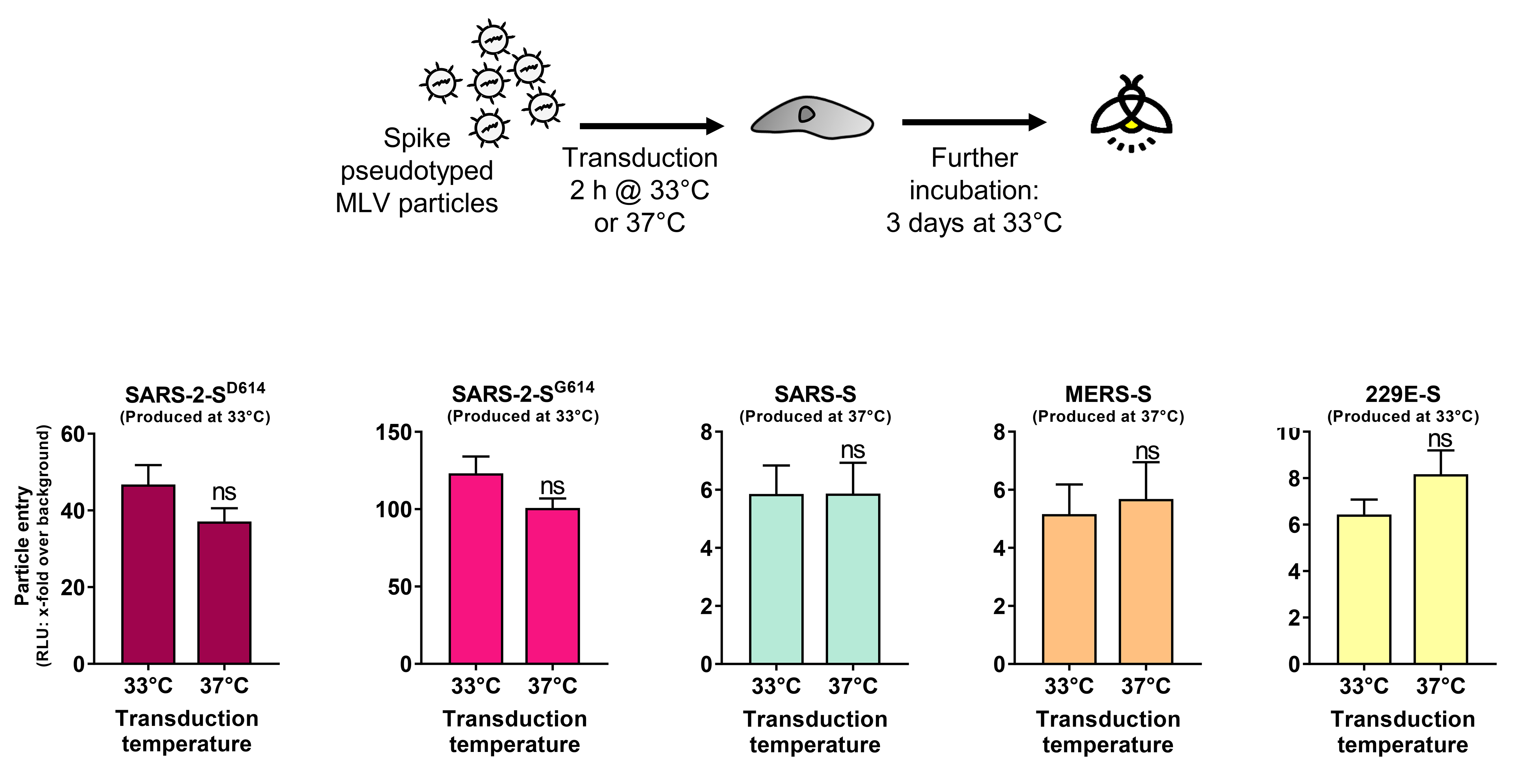

Supplement: S2 Fig — S-pseudotyped MLV particles were produced at their optimal production temperature (33°C or 37°C), harvested and used for transduction of HEK293T target cells expressing the appropriate receptor and TMPRSS2. Transduction was carried out for 2 h at either 33°C or 37°C, after which particles were removed, fresh medium was added and further incubation was done at 33°C. At day 3 post transduction, particle entry was measured by luminescence read-out (mean ± SEM from three experiments). ns, P > 0.05 (two-tailed unpaired t-test, 37°C versus 33°C). (TIF) [file ppat.1009500.s006.tif]

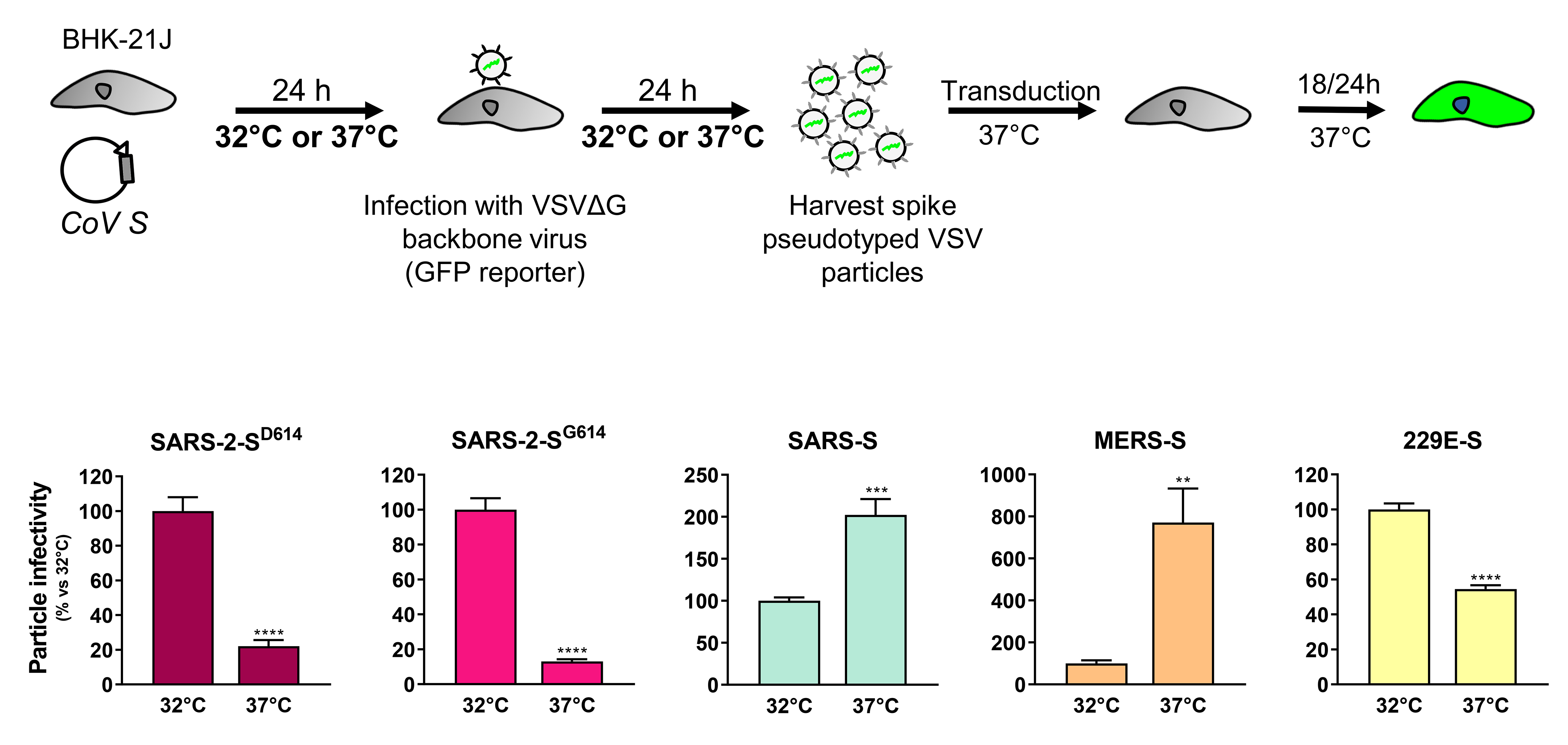

Supplement: S3 Fig — GFP-encoding pseudoviruses bearing different S-proteins were produced in BHK-21J cells at either 32 or 37°C. Next, they were transduced into target cells, i.e. Vero E6 for SARS-S- and SARS-2-S-bearing pseudoviruses; Huh-7 for MERS-S; or 16HBE for 229E-S, and incubated at 37°C. One day later, the number of GFP-expressing cells was quantified by high-content imaging. N = 2, performed in triplicate. **, P ≤ 0.01; ***, P ≤ 0.001; ****, P ≤ 0.0001 (two-tailed unpaired t-test; 37°C versus 32°C). (TIF) [file ppat.1009500.s007.tif]

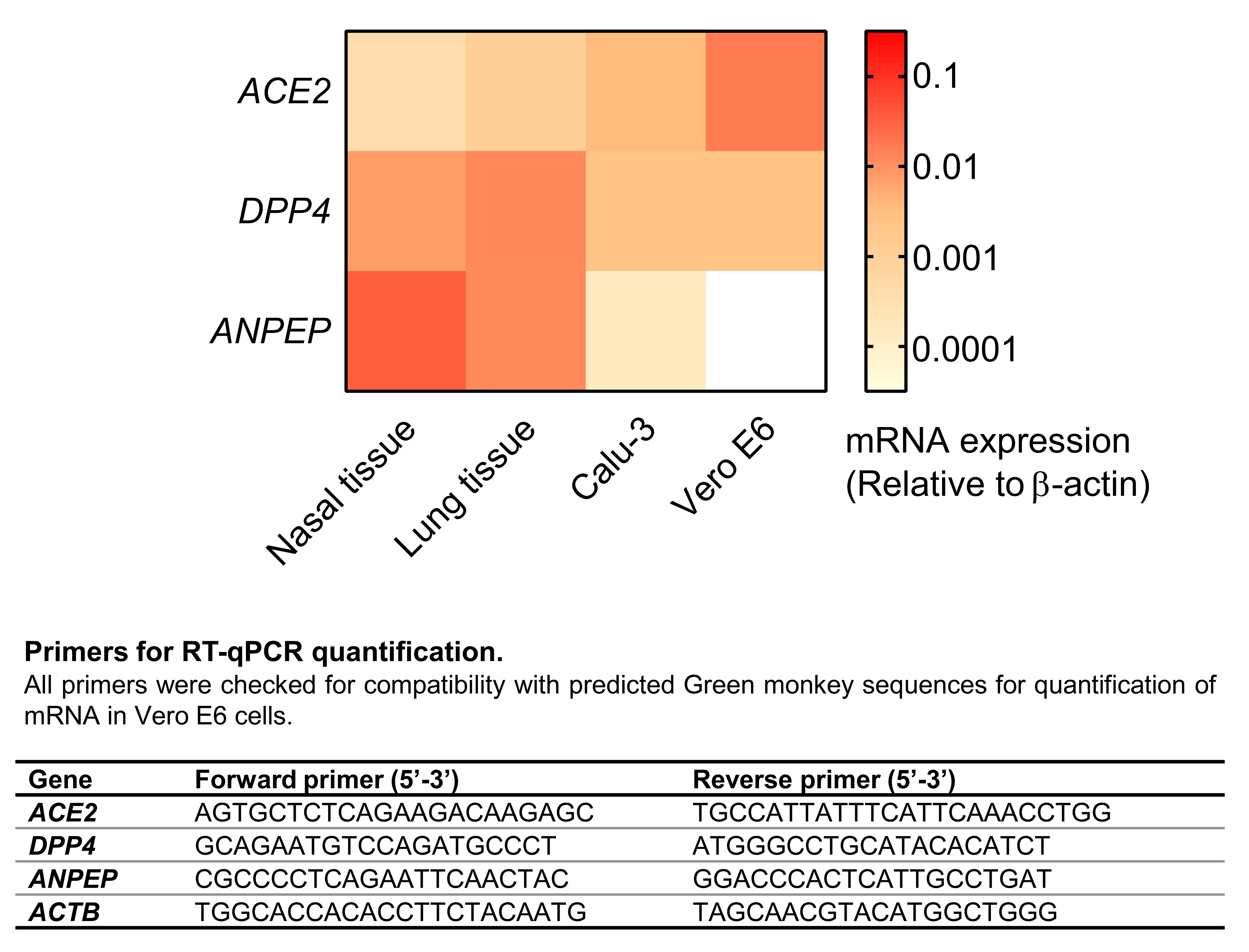

Supplement: S4 Fig — The heatmap shows mRNA levels of the receptor transcripts (relative to β-actin), determined by RT-qPCR. The Table shows the primer sequences used for RT-qPCR analysis. (TIF) [file ppat.1009500.s008.tif]

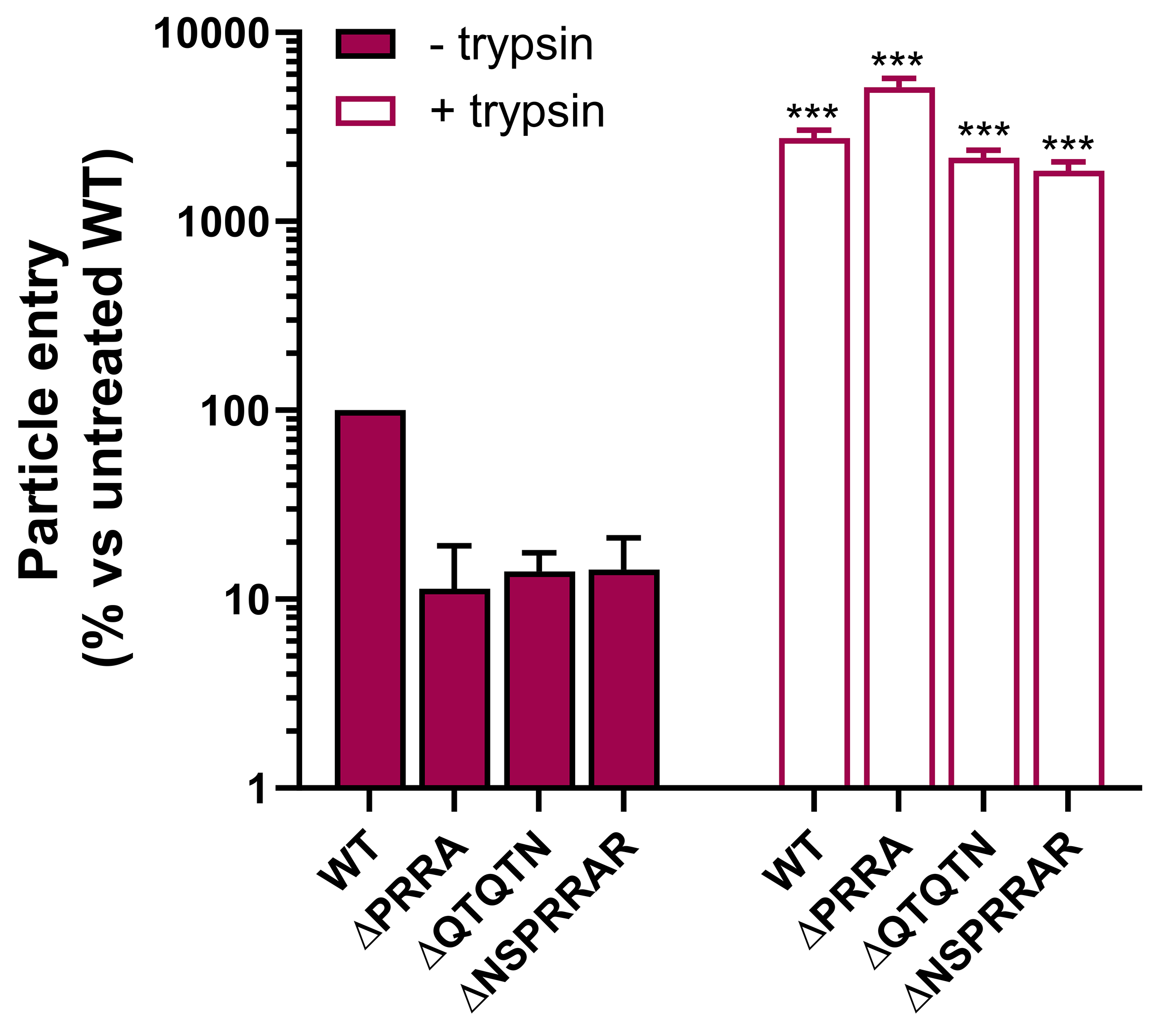

Supplement: S5 Fig — The pseudoparticles were allowed to bind to Calu-3 cells for 1 h at 4°C, after which unbound particles were removed and DMEM with 10 μg/ml TPCK-trypsin was added. After 2 h at 37°C, the medium was replaced by Calu-3 growth medium. Results are the mean ± SEM; N = 3. ***, P ≤ 0.001 (two-tailed unpaired t-test; trypsin-treated versus -untreated condition). (TIF) [file ppat.1009500.s009.tif]
